# Elastic interaction between Mauna Loa and Kīlauea evidenced by independent component analysis

**DOI:** 10.1038/s41598-022-24308-0

**Published:** 2022-11-18

**Authors:** Monika Przeor, Luca D’Auria, Susi Pepe, Pietro Tizzani, Iván Cabrera-Pérez

**Affiliations:** 1 Instituto Volcanológico de Canarias (INVOLCAN), San Cristóbal de La Laguna, Spain; 2 Instituto Tecnológico y de Energías Renovables (ITER), Granadilla de Abona, Spain; 3 Istituto per il Rilevamento Elettromagnetico dell’Ambiente (IREA-CNR), Napoli, Italy

**Keywords:** Geophysics, Volcanology

## Abstract

The contrasting dynamics between Mauna Loa and Kīlauea have been studied over the last 100 years from multiple viewpoints. The fact that dynamic changes of one volcano trigger a dynamic response of the other volcano indicates a connection may exist. Petrological works show a direct relationship between the magmatic systems of these two volcanoes is not possible. We analysed DInSAR data and GPS measurements of ground deformation patterns associated with the activity of Mauna Loa and Kīlauea volcanoes. The DInSAR SBAS dataset spans the interval between 2003 and 2010, and was acquired along ascending and descending orbits of the ENVISAT (ESA) satellite under different look angles. Of the 10 tracks that cover the Big Island (Hawai‘i), 4 cover both volcanic edifices. Using GPS measurements, we computed the areal strain on 15 triplets of stations for Kīlauea volcano and 11 for Mauna Loa volcano. DInSAR data was analysed by applying Independent Component Analysis (ICA) to decompose the time-varying ground deformation pattern of both volcanoes. The results revealed anticorrelated ground deformation behaviour of the main calderas of Mauna Loa and Kīlauea, meaning that the opposite response is seen in the ground deformation of one volcano with respect to the other. At the same time, Kīlauea exhibits a more complex pattern, with an additional component, which appears not to be correlated with the dynamics of Mauna Loa. The GPS areal strain time series support these findings. To corroborate and help interpret the results, we performed inverse modelling of the observed ground deformation pattern using analytical source models. The results indicate that the ground deformation of Mauna Loa is associated with a dike-shaped source located at 6.2 km depth. In comparison, the anticorrelated ground deformation of Kīlauea is associated with a volumetric source at 1.2 km depth. This excludes a hydraulic connection as a possible mechanism to explain the anticorrelated behaviour; instead, we postulate a stress-transfer mechanism. To support this hypothesis, we performed a 3D numerical modelling of stress and strain fields in the study area, determining the elastic interaction of each source over the others. The most relevant finding is that the Mauna Loa shallow plumbing system can affect the shallowest magmatic reservoir of Kīlauea, while the opposite scenario is unlikely. Conversely, the second independent component observed at Kīlauea is associated to a sill-shaped source located at a depth of 3.5 km, which is less affected by this interaction process.

## Introduction

The interaction processes between the two most active Hawaiian volcanoes are still controversial, and despite multiple studies carried out over more than a century, an unambiguous model has yet to be identified. In order to provide new insights to this discussion, we compared the ground deformation patterns in both volcanoes using DInSAR SBAS and Global Positioning System (GPS) datasets. In this work, we processed 10 tracks of ENVISAT ASAR satellite images from 2003-2010, together with available GPS data from 15 stations located around the two summit calderas of Mauna Loa and Kīlauea. We applied the Independent Component Analysis (ICA) to the DInSAR SBAS ground deformation data to reveal relationships between the spatio-temporal patterns of the ground deformation of the two volcanoes. ICA is widely used Data Mining technique, which allows detecting, separating and characterizing hidden patterns into a spatio-temporal dataset^1^. Furthermore, we computed the GPS areal strain time series around Mauna Loa and Kīlauea calderas, comparing them with the results provided by ICA. Subsequently, we present inverse modelling of ground deformation sources, which provides constraints for conceptual models of the shallow feeding system of Mauna Loa and Kīlauea. Conclusively, we realized a 3D numerical modelling of the stress and strain fields produced by the inflation/deflation of the individual ground deformation sources to better understand the mechanism of their interaction. The details of data processing and modelling are described in the Methods section.

**Figure 1:**
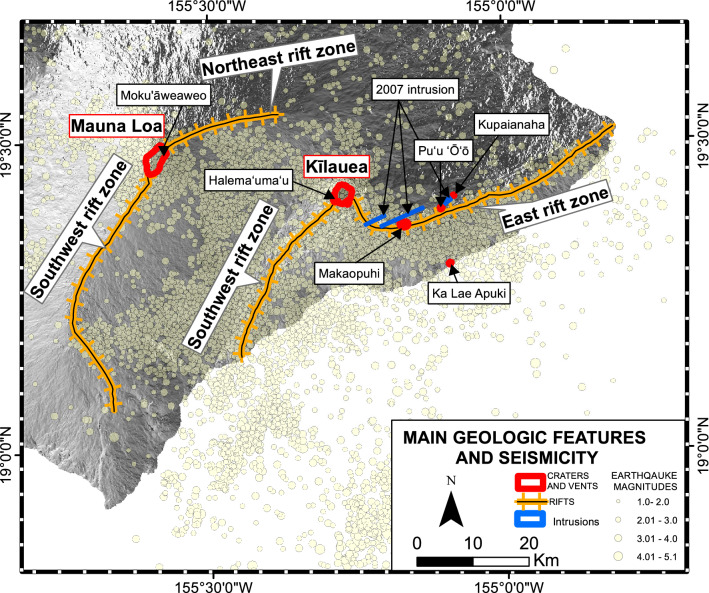
Main geologic and seismicity map of Mauna Loa and Kīlauea^3^.

Hawai‘i Island is composed of 5 shield volcanoes: Kohala, Mauna Kea, Hualālai, Kīlauea, and Mauna Loa, with the latter being the largest active volcano on Earth. It has erupted 35 times since its first historical eruption in AD 1750^2^. The summit area of Mauna Loa is composed of a large summit caldera (Moku’āweoweo) and two elongated ridges: the Northeast Rift Zone (NERZ) and the Southwest Rift Zone (SWRZ); (Fig. 1). Kīlauea volcano overlaps the southeastern flank of Mauna Loa. It has been in near- continuous eruption from 1983 to 2018^2^, and its main volcano-tectonic features are the East Rift Zone (ERZ), the Southwest Rift Zone (SWRZ), a large summit caldera, and the Halema’uma’u crater within it^2^ (Fig. 1).

Recently, several studies have aimed to characterise the nature of the magmatic source responsible for ground deformation at Kīlauea and Mauna Loa volcanoes^3–17^. The two principal sources at Mauna Loa volcano are associated with shallow dike intrusion into the central conduit of Mauna Loa, and its rift zones^14^. Dike intrusions create compression over the adjacent flanks of the volcano^18,19^ and consequently, they produce ground deformation and earthquakes along the basal decollement zone^20^. In the case of Kīlauea volcano, the general picture is similar to Mauna Loa: the main ground deformation sources are linked to the central feeding system as well as to magmatic intrusions along the rift zones^7^ that are also responsible for the seismicity near the base of the volcano^21,22^. The intrusion of dikes and extension of the rift-zone^9^ also causes the shortening at the base of the edifice and the uplift along the frontal bench^8,23^.

At Mauna Loa, Amelung et al.^14^ identified a spheroidal source reservoir beneath the southeast margin of Moku’āweoweo Caldera, connected to an elongated source linked to the rift zones. Pepe et al.^5^, showed that the main source of ground deformation at Mauna Loa consists of a vertical pipe connected to dike-shaped reservoirs located along the rift zones.

Poland et al.^3^ showed the existence of various magmatic reservoirs beneath Kīlauea: one below the caldera of Halema‘uma‘u, the Keanakāko‘i reservoir, the South Caldera reservoir, and both Rift Zones (East Rift Zone and Southwest Rift Zone). The Halema‘uma‘u reservoir is the summit storage located between 1 and 2 km depth below the main caldera. The Keanakāko‘i is considered a temporal storage, with magma inputs occurring intermittently. The South Caldera reservoir, mentioned by Poland et al.^3^ is located at a depth of 3–5 km below the Halema‘uma‘u caldera and is considered the principal storage of magma at Kīlauea. Both Rift Zones are a set of fractures and vents with directions toward the East and Southwest of the main caldera. A full catalogue of dike intrusions over Kīlauea edifice can be found in Montgomery–Brown et al.^24^.

In the last three decades, some relevant deformation episodes took place at Kīlauea and were linked to the Pu’u ’Ō’ō-Kūpa’ianahā eruption (1983–2018)^3–17,25^. Volcanic activity at Pu’u ’Ō’ō-Kūpa’ianahā from 1983 to 2001 was characterised by dominant deflation, followed by a new inflation episode starting in 2001 when Kīlauea experienced a new uplift phase. Six months later, similar behaviour at Mauna Loa volcano was observed^13^. The enhancement of volcanic activity in this period was caused by an increase in the magma supply from the mantle^13^.

The sudden inflation that started in 2003 at Kīlauea mainly affected the summit caldera, but was recorded as far away as 50 km from the summit and lasted until 2007^17^. In 2005, magma accumulation in ERZ led to summit inflation and an increase in the output of $SO_2$^13^. In the same year, a major collapse of the lava delta occurred^15^, and one year later, an uplift episode along the southern part of the Kīlauea summit caldera was registered^15^. The ground deformation pattern of Kīlauea during 2003–2007 was dominated by inflation along the ERZ and the summit crater^17^.

In this work, we consider only the magmatic intrusions that occurred in the summit area of the volcanoes and only during the time interval considered in this study. A major episode of volcanic unrest occurred between 2003-2010, beginning on 17th June 2007 (American Father’s Day 2007, FD07). The event entailed changes in volcanic activity and formation of new eruptive vents^17^. It caused rapid deflation of the Kīlauea summit area due to magma withdrawal to eruptive vents located along the ERZ, about 8 km away^15–17^. The first episode of the FD07 eruption lasted for two days. On 21st July of 2007, another eruptive episode began along the ERZ, with vents located about 19 km away from the summit^15,16^. ERZ vent activity was then continuous, while the summit crater of Halema’uma’u showed increased volcanic gas emission levels until an explosion of Kīlauea’s main summit crater on 19th March 2008^17^. After the FD07 episode until 2008, the ground deformation pattern of Kīlauea was characterised by deflation, and summit seismicity returned to background values^17^.

The interaction between the two most active volcanoes of Hawai‘i Island has been discussed for over 100 years^11,12,26^. Rhodes and Hart^27^ confirm that the chemical composition of lavas at Kīlauea and Mauna Loa are different, indicating the magmatic feeding systems are independent (at least at the crustal level). However, geophysical studies seem to indicate the opposite: Klein^11^ first noticed the anticorrelation between the two volcanoes, emphasising that an increase in activity at Kīlauea often corresponds to a decrease in Mauna Loa dynamism. Miklius and Cervelli^12^ captured the opposite behaviour in the ground deformation patterns of the volcanoes: at the beginning of the high-volume effusive episode in Kīlauea, inflation of Mauna Loa was observed (May 2002). Shirzaei et al.^28^ studied the coupling behaviour of both volcanoes between 2003 and 2008. The authors postulate that the causative source of the interaction between the two volcanoes is related to deep-seated mantle surges. Despite being the subject of many studies, the nature and the mechanism of the interaction between the feeding systems of the two volcanoes and their level of interconnection are still contentious and remain unclear.

## Results

**Figure 2:**
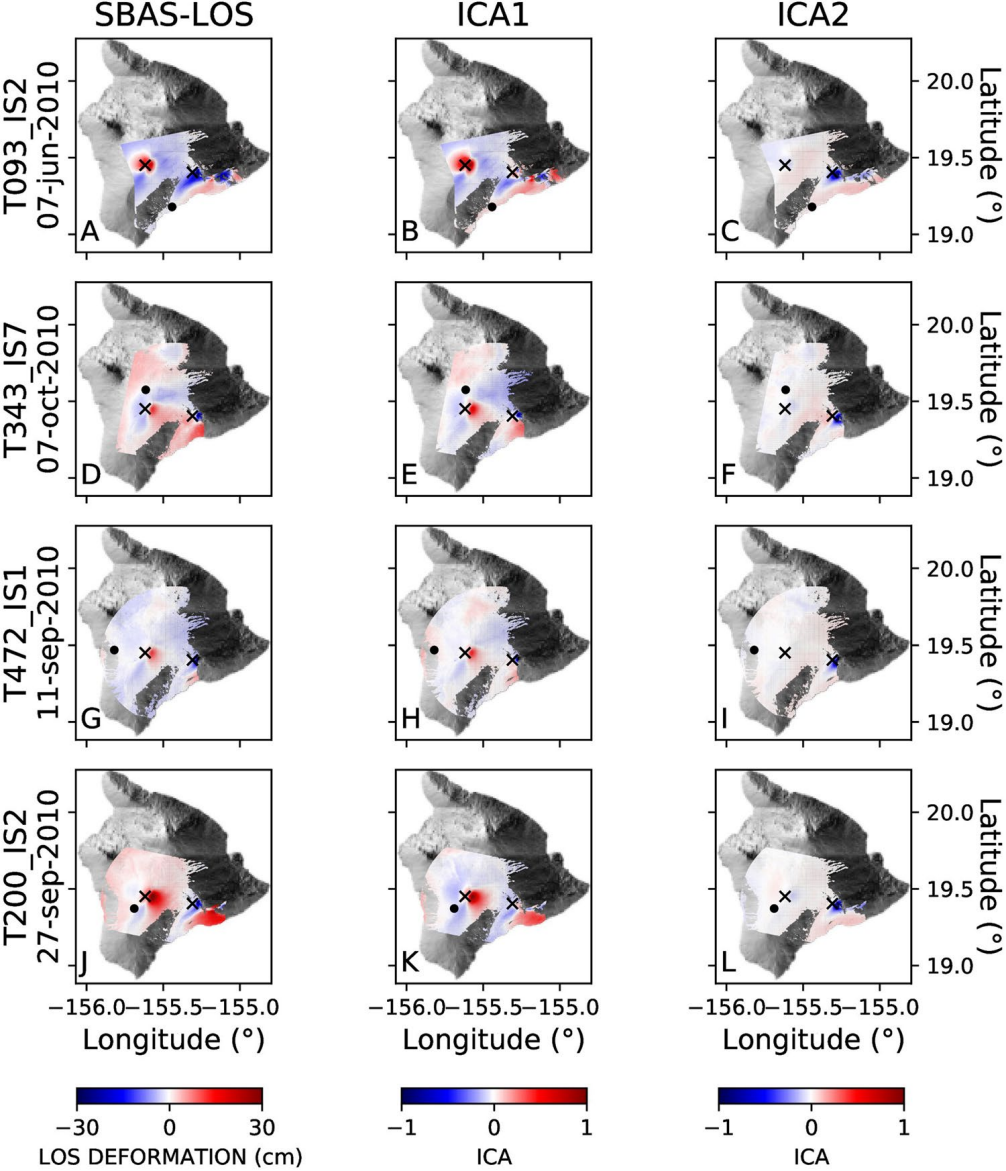
LOS deformation cumulative maps. (**A**), (**B**), (**C**): ENVISAT 093 orbit; (**D**), (**E**), (**F**): ENVISAT 343 orbit; (**G**), (**H**), (**I**) : ENVISAT 472 orbit; (**J**), (**K**), (**L**): ENVISAT 200 orbit (see Table S2 for track details). Column 1 shows the LOS cumulative displacement DInSAR map; columns 2 and 3 represent the first and second components resulting from applying the ICA decomposition algorithm. The black crosses indicate the points used for extracting the time series shown in Fig. 3, while black dots are the reference points used for the DInSAR SBAS processing.

**Figure 3:**
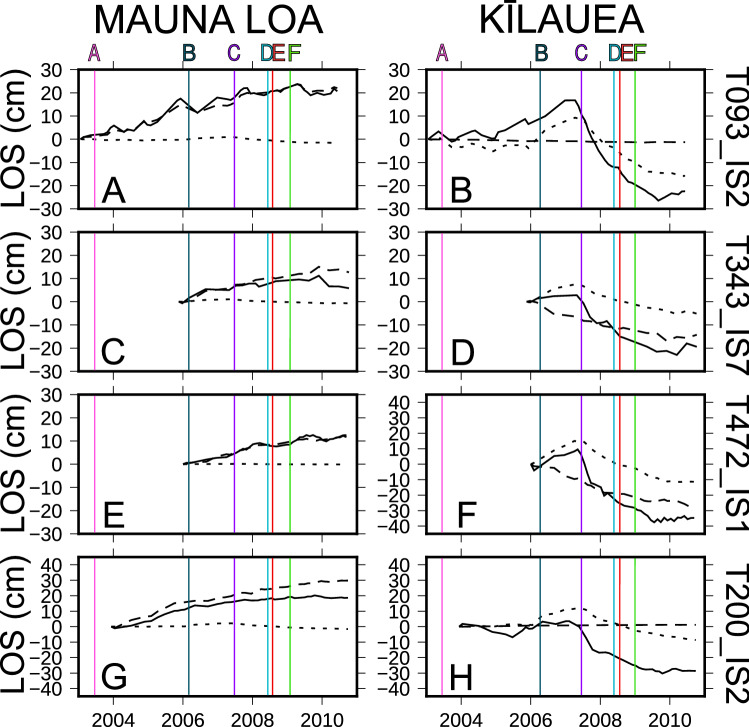
LOS displacement time series of Mauna Loa and Kīlauea summits. (**A**), (**C**), (**E**), (**G**): time series for LOS displacement of Mauna Loa; (**B**), (**D**), (**F**), (**H**): time series for Kīlauea volcano. Time series of LOS data are shown with continuous black lines; time series of the first component of ICA are indicated with the dashed black lines, while the dotted black lines report the time series of the second component of ICA. Vertical lines mark major volcanic episodes and the date of the beginning of the GPS dataset: (**A**) Mother’s Day flow, which began erupting from Pu’u ’Ō’ō on 12th May 2003; (**B**) small bench collapse on 30th July 2006; (**C**) Father’s Day eruption on 17th June 2007; (**D**) explosion on 19th March 2008; (**E**) the second half of 2008 which represent the time interval where the GPS measurements are available; (**F**) active vent within Halema’uma’u crater in January 2009.

The analysis of the observed ground deformation, reported in the first column of Fig. 2(A, D, G, J), suggests an anticorrelated behaviour of the ground deformation between Mauna Loa and Kīlauea volcanoes. This is clearly seen in the normalized spatial patterns (indicated as $B_k$ in Eq. 1) of the first component (ICA1), and shown in Fig. 2. The anticorrelation between these two volcanoes is demonstrated by the presence of positive values on Mauna Loa and negative values on Kilauea on the ICA1 component for all four selected tracks. The anticorrelation between the two volcanoes is seen only by the ICA1 component on both Mauna Loa and Kīlauea. The temporal variation is shown in Fig. 3 with dashed lines, clearly showing the opposite behaviour of Mauna Loa and Kīlauea on all four tracks: when ground deformation of Mauna Loa shows positive values, Kīlauea exhibits negative ones. It should be noted that the sign of the ICA components is arbitrary, being the actual value of the ground deformation modulated by the coefficients $A_jk$ in Eq. (1)1.

Also, let us remark that, as shown by Fig. 2, there are other evidences of the ground deformation far from their summit areas. Column 1 in Fig. 2 shows an example of such deformation in the southeast side of the Kīlauea volcano associated with the faults system mentioned by Shirzaei et al.^29^.

The second component of the ground deformation pattern (ICA2 in Fig. 2) exhibits significant values only in the area of Kīlauea volcano. The overall shape (Fig. 2) and temporal behaviour (Fig. 3) of the two components are different. The maximum of the ICA2 component at Kīlauea is located on the southern side of the summit caldera, slightly displaced to the south with respect to the maximum of the ICA1 component on the same volcano.

The above-mentioned anticorrelated behaviour in the ground deformation pattern is visible also by considering the LOS deformation time series. In Fig. 3, the time series for two selected pixels are shown, which correspond with the summit calderas of Mauna Loa and Kīlauea. The Mauna Loa time series displays an uplift from 2003 until late 2009. Simultaneously, the Kīlauea shows an inflation pattern until 2007 (the FD07 volcanic episode), followed by significant subsidence until late 2009.

At Mauna Loa, the total ground deformation pattern is almost exclusively represented by the first ICA component for all four considered tracks (see panels A, C, E, and G in Fig. 3). Minor differences between the ICA1 and the total LOS on this volcano can be attributed to a noisy component that ICA cannot model. In the case of Kīlauea volcano (see panels B, D, F, and H in Fig. 3), the contribution of both components is relevant. The anticorrelation in the temporal pattern of ICA1 is seen by a monotonic increase for Mauna Loa (ICA1, Fig. 3), which corresponds to a monotonic decrease in Kīlauea (seen on tracks T343 and T472; Fig. 3, dashed line). This difference in the amplitude of the ICA1 on the four considered tracks on Kīlauea can be attributed to the different orbits or, in other words, to the different LOS directions of the considered tracks. Conversely, the second ICA component displays a more complex temporal pattern, with an increase until the FD07 eruption, followed by a decreasing trend until the end of 2009.

The GPS data analysis reveals similar patterns to the SBAS time series for both volcanoes (Figs. S1 and S2 in supplementary material). The vertical axis is in order of 5 and 10 µstrain, respectively. Mauna Loa volcano displays slow strain rates until the second part of 2009, when the rate increases significantly in triplets of stations located in the southeast part of the summit caldera. Although the last four triplets (those located in the SE part of the main crater) dominantly see the decrease in strain area that began in mid-2009, the other triplets of stations show a smaller but evident decrease too. From mid-2008 to mid-2009, the strain area of Kīlauea diminished (Fig. S2). After the second half of 2009, GPS stations in Kīlauea crater and its surroundings record a significant increase in the strain area.

The GPS ground deformation patterns show the different behaviour of Mauna Loa and Kīlauea (Figs. S1 and S2). Specifically, since 2009, the patterns of the two volcanoes show an opposite behaviour. This agrees with the results of ICA decomposition, shown in Fig. 3. The ICA2 component does not vary much since mid-2009; hence the ground deformation is shown in the anticorrelated ICA1 pattern. This does not hold for the previous interval, where ICA2 is prevalent on the ground deformation at Kīlauea.

The ICA analysis of the DInSAR SBAS time series of the satellite tracks highlight the presence of an anticorrelated ground deformation pattern linked to at least two sources located beneath the summit calderas of Mauna Loa and Kīlauea (component ICA1). Furthermore, the presence of another source is evidenced by the ICA2 component beneath Kīlauea alone. In order to better understand the physical mechanisms responsible for the observed ground deformation patterns, we performed inverse modelling of the three detected sources and used Akaike Information Criterion (AIC)^30^ to select the appropriate model for each source.

**Figure 4:**
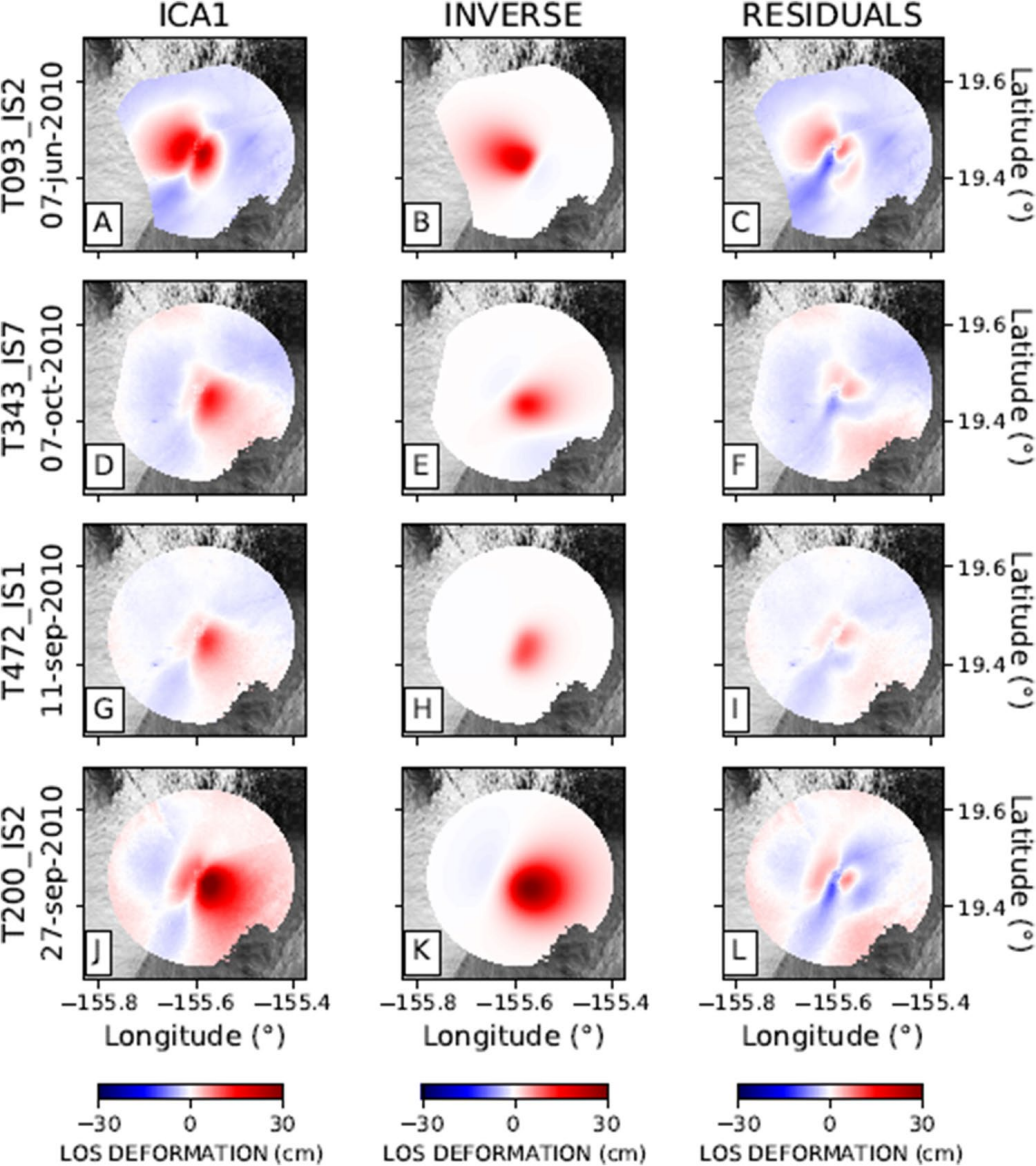
Inverse modelling of the Mauna Loa ground deformation source for four selected tracks: (**A**), (**B**), (**C**): ENVISAT 093 orbit; (**D**), (**E**), (**F**): ENVISAT 343 orbit; (**G**), (**H**), (**I**): ENVISAT 472 orbit; (**J**), (**K**), (**L**): ENVISAT 200 orbit, respectively. Column 1 represents the first component (ICA1) encompassing a radius of 20 km from Moku’āweoweo Crater. Column 2 represents the inverse model. Column 3 shows the residuals.

**Figure 5:**
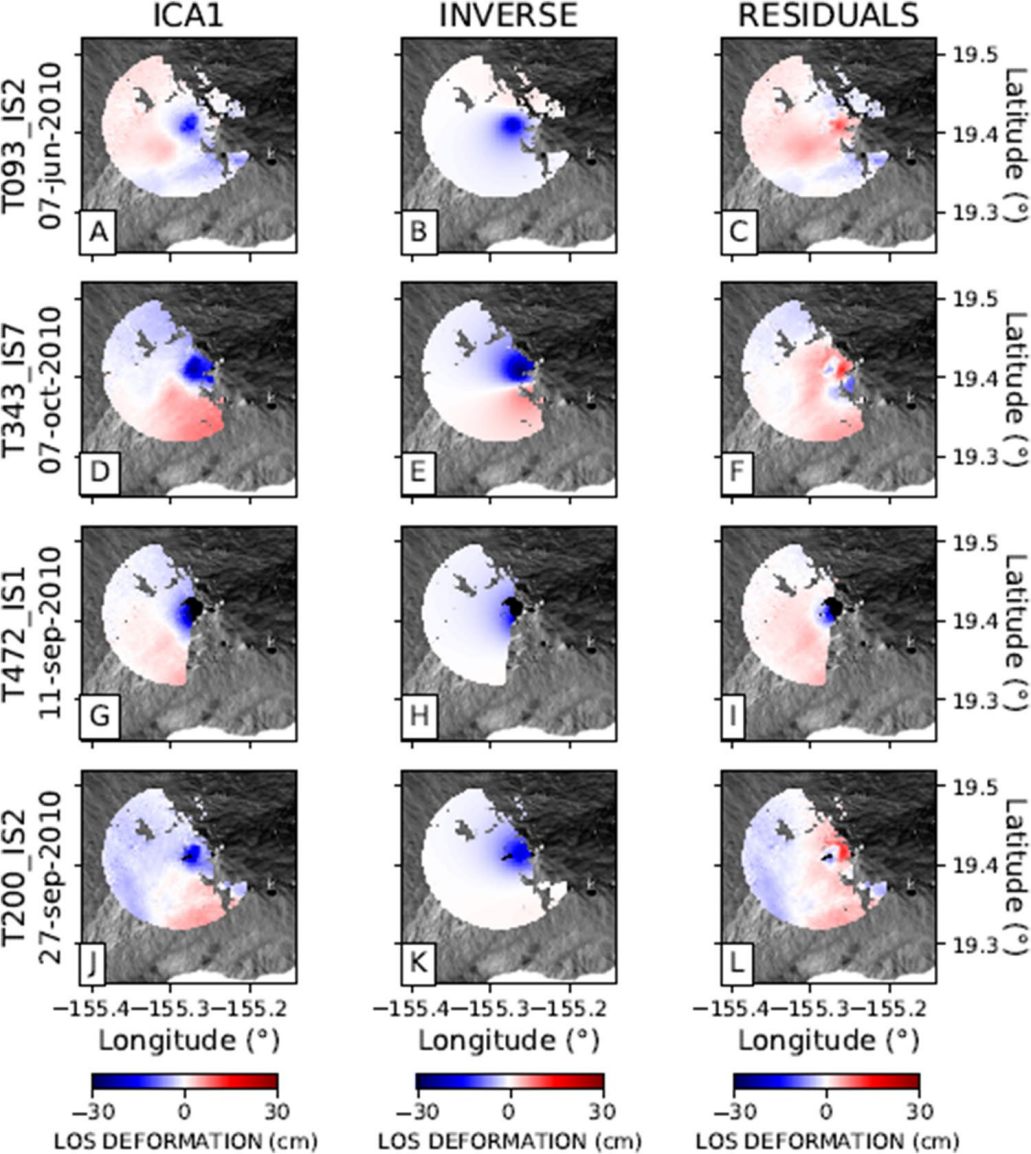
Inverse modelling of the Kīlauea ground deformation source for four selected tracks: (**A**), (**B**), (**C**): ENVISAT 093 orbit; D, E, F: ENVISAT 343 orbit; G, H, I: ENVISAT 472 orbit; J, K, L: ENVISAT 200 orbit, respectively. Column 1 represents the first component (ICA1) encompassing a radius of 11 km from Halema’uma’u Crater. Column 2 represents the Mogi analytical model as a result of the inverse modelling. Column 3 shows the residuals of the inverse modelling and the observed data.

**Figure 6:**
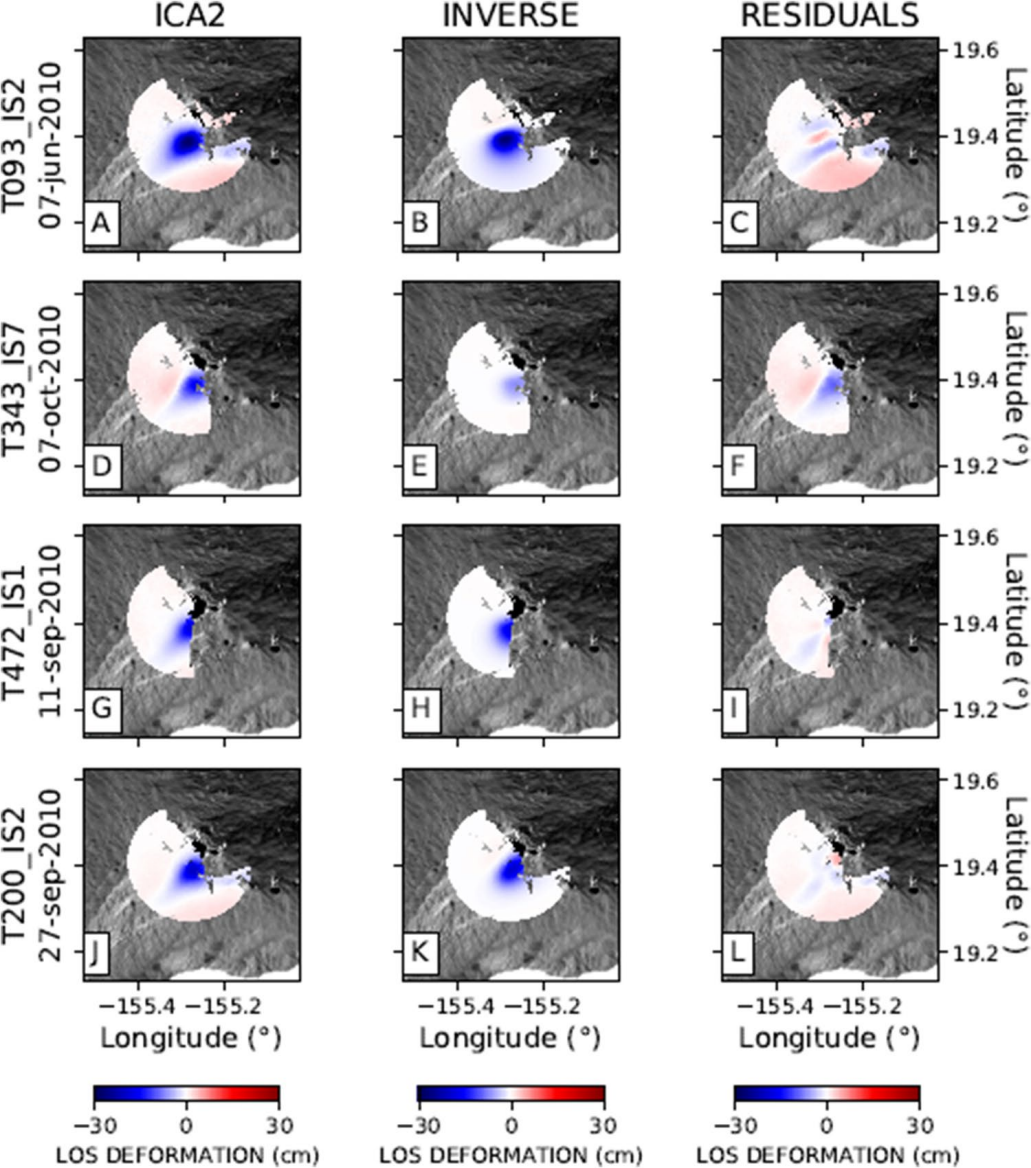
Inverse modelling of the Kīlauea ground deformation source for four selected tracks: (**A**), (**B**), (**C**): ENVISAT 093 orbit; (**D**), (**E**), (**F**): ENVISAT 343 orbit; (**G**), (**H**), (**I**): ENVISAT 472 orbit; (**J**), (**K**), (**L**): ENVISAT 200 orbit, respectively. Column 1 represents the first component (ICA2) encompassing a radius of 15 km from Halema’uma’u Crater. Column 2 represents the inverse model. Column 3 shows the residuals.

In Table S1 in supplementary material, we detail the parameters of the three retrieved sources. Based on AIC, the temporal variation of Mauna Loa area displacements (delineated by ICA1) are best explained by a sub-vertical Okada crack model, with a centroid located at 6.2 km depth (Fig. 4; Table S1). The ICA1 component for Kīlauea is better represented by a simple Mogi^31^ source located at 1.2 km depth (Fig. 5). The ICA2 component for Kīlauea is best described by a sub-horizontal Okada^32^ crack located at 3.5 km depth (Fig. 6; Table S1 in supplementary material).

Figures 4, 5, and 6 show the inverse modelling results for each of the three modelled sources of ground deformation. Each Figure shows the spatial pattern of the ground deformation associated with the relevant ICA, the best-fit analytical model, and the residuals.

**Figure 7:**
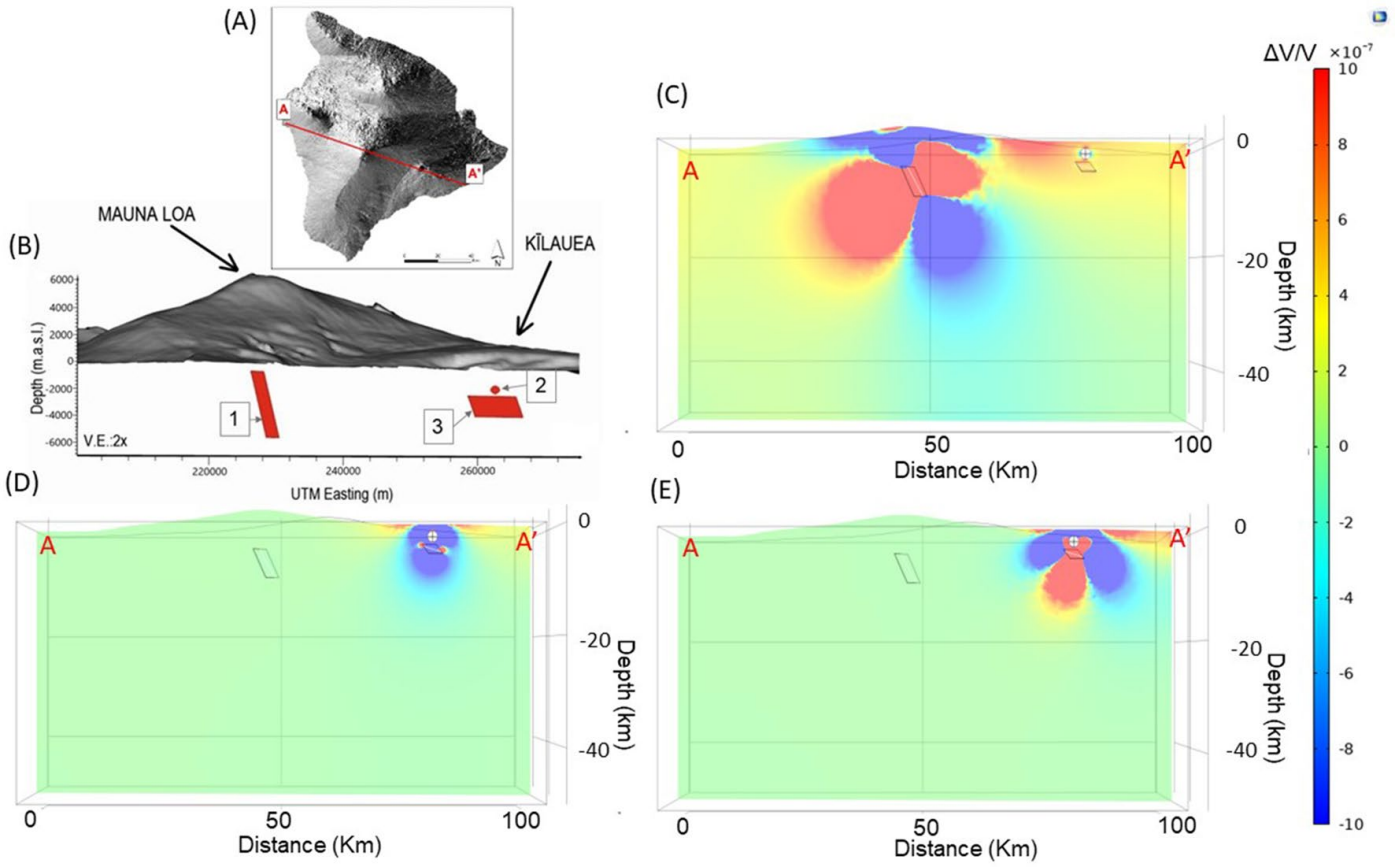
3D numerical modelling of strain fields. (**A**) The Digital Elevation Model of Hawai’i Island, with the red line representing the trace (A-A′), used in panels (**C**–**E**). (**B**) tridimensional representation of the modelled ground deformation sources of Mauna Loa and Kīlauea volcanoes. The sources are numbered according to Table S1 in the supplementary material. (1) the Mauna Loa dike-shaped source from ICA1; (2) the Kīlauea Mogi-like source from ICA1; (3) the Kīlauea sill-shaped source from ICA2. Storage areas and topography are exaggerated in size for clarity. (**C**) the volumetric strain field represented along the A-A′ trace due to an overpressure applied to the Mauna Loa dike-like source. (**D**) volumetric strain field along the A-A′ trace due to an overpressure applied to the Kīlauea Mogi-like source. (**E**) volumetric strain field along the A-A′ trace due to an overpressure applied to the Kīlauea sill-like source.

The panel (B) in the Fig. 7, shows a schematic tridimensional perspective of the three sources of ground deformation resulting from inverse modelling. Beneath Mauna Loa volcano, there is an Okada dike-shaped source. In the area of Kīlauea, there are Mogi-like and Okada sill-shaped sources of deformation.

## Discussion

Previous studies of Mauna Loa and Kīlauea hypothesised the presence of a connection between these two volcanoes. In this context, Kīlauea volcano provided considerable geophysical and geochemical data due to its constant activity from 1983 to 2018. However, the lower density of the geophysical monitoring network of Mauna Loa with respect to Kīlauea, poses some difficulties in comparing the geophysical data of the two volcanoes^3^. This work has provided evidence for two sources associated with ICA1 and showing an anticorrelated temporal relationship between the Mauna Loa and Kīlauea (Fig. 3).

The findings of the inverse modelling are consistent with previous studies. Poland et al.^3^ postulated the existence of two long-term magma reservoirs beneath the Kīlauea summit. Both reservoirs are connected to the rift zone system. Additionally, there exists a temporary storage area beneath Keanakaoko’i Crater. They highlighted the presence of a shallower magma reservoir located in the eastern margin of Halema’uma’u Crater, at a depth of 1–2 km depth. This source may coincide with the Kīlauea-ICA1 source proposed here, located at 1.2 km depth (Fig. 5; Table S3). According to Poland et al.^3^, deeper magma storage at Kīlauea is situated at about 3 km depth and displaced to the south of Halema’uma’u Crater. This is consistent with our inversion results, which show a sill-shaped source located at a 3.5 km depth. The inverse model of the second and deeper source of the Kīlauea volcano is given in Fig. 6, with parameters detailed in Table S1 in supplementary material. Our results for the Kīlauea feeding systems are compatible with Poland et al.^3^ showing the presence of at least two ground deformation sources active between 2003 and 2010. However, Poland et al.^3^ postulate the existence of a spherical or an ellipsoidal source in the southern side of the main caldera of Kīlauea. In contrast, our inversion results indicate the existence of a sill-like reservoir. Let us remark that our source model is related only to the ground deformation pattern related to the second component (ICA2) of decomposition. We again emphasise the effectiveness of ICA in separating the contribution of individual sources, significantly reducing the intrinsic ambiguity in geodetic inverse problems.

At Mauna Loa, previous studies emphasised the relevance of the rift zones as sources of ground deformation^4,5^. Our findings suggest a dike-like geometrical structure as a primary source during the interval considered (Fig. 4). During our analysed period (2003–2010), the ICA decomposition detected only one component of ground deformation in Mauna Loa. This is in agreement with Pepe et al.^5^, who, although proposing a more complex geometrical model, showed that the main reservoir and the rift zone acted with synchronous behaviour.

Many authors have already suggested possible connections between Mauna Loa and Kīlauea. Miklius and Cervelli^12^ proposed a crustal-level interaction between them: pulses of magma in the plumbing system of Mauna Loa may cause pressure variations in the Kīlauea shallow magma system. Gonnermann et al.^13^ explained observed related ground deformation patterns by considering a pore-pressure diffusion within a thin accumulation layer in the asthenosphere. Since the magma composition at Kīlauea is isotopically distinct from Mauna Loa^27^, Gonnermann et al.^13^ suggested the interconnection between Mauna Loa and Kīlauea must be explained by the transfer of stress by pore-pressure variations. Shirzaei et al.^28^ explain the interconnection between the Mauna Loa magma chamber and the Kīlauea rift zone through pore pressure diffusion in an asthenospheric magma supply system.

We note that volcanic interconnection is even more evident if we consider the temporal evolution of the ground deformation patterns. From 1983 until 2003, both volcanoes were deflating^12,14–17^. The time series of DInSAR data (Fig. 3) shows the change of the ground deformation that took place in 2003 and was explained by many authors as sudden inflation that started in Kīlauea and lasted until 2007. That inflation resulted in an eruption that started in the northern part of Makaopuhi Crater due to higher magma rates in ERZ^3^. While Kīlauea showed higher volcanic activity accompanied by ground deflation mostly represented by the continuous and dotted line in Fig. 3 in Kīlauea block, Mauna Loa was still experiencing gradual inflation of the ground - continuous and dashed line in Mauna Loa block (Fig. 3). Many authors have attempted to explain these opposing ground deformation pattern behaviours, presenting models for the causes. Poland et al.^17^ stated that the 2003-2007 episode was an unusual event, caused by the arrival of a new pulse of magma from the mantle. Dzurisin et al.^33^ found similar behaviour in the late 70s: when Kīlauea experienced an increase in magma supply, inflation of Mauna Loa was also observed. Poland et al.^3^ explained this similar behaviour by magma input to the deep storage zone that affected both volcanoes.

The DInSAR SBAS time series analysis via the ICA decomposition technique provides a powerful tool for assessing and highlighting the relationship between the magmatic systems of the two volcanoes, and can be used to constrain the underlying physical mechanism of their possible connection. Interconnection between the two volcanic systems is best shown by the first independent component of the ground deformation (ICA1). In contrast, ICA2 shows a ground deformation source affecting only Kīlauea volcano. The rapid deflation of Kīlauea from 2007 to 2009 (Fig. 3) has a source located beneath Halema’uma’u Crater. Co-incident with deflation of this source, the interconnected source of Kīlauea and Mauna Loa (ICA1) was deflating more gradually (Fig.3).

The GPS strain area results support anticorrelated behaviour of these two volcanoes. The 2009 strain area reduction in Mauna Loa and the opposite increment of the strain area in Kīlauea indicates that while Mauna Loa was undergoing a deflation, Kīlauea was experiencing an inflation episode.

Finally, based on the inverse modelling results, we maintain that the pattern associated with the anticorrelated component ICA1 points to a very shallow source beneath Kīlauea. This makes it unlikely that there is a direct connection between the magmatic systems of the two volcanoes in the deeper crust. A direct connection at shallow depths would provide a further source of ground deformation, which has never been observed. Furthermore, it would contradict petrological and geochemical evidence^27^. On the other hand, the existence of a second component, related to Kīlauea alone, suggests a more complex configuration of the plumbing system of Kīlauea, characterised by multiple reservoirs.

The spatial configuration suggests a possible explanation for the observed anticorrelation between the ground deformation sources related to ICA1 (numbered 1 and 2 in Fig. 7). Their respective geometries make the stress field, caused by the inflation of one source, act on the other with forces directed inward along its external surface. At the same time, because of its sill-like geometry, the source related to ICA2 (number 3 in Fig. 7) would be less sensitive to these changes in the stress field, explaining its independent temporal behaviour. In practice, the dike-shaped source of Mauna Loa would be capable of affecting the shallow volumetric (Mogi-like) source of Kīlauea, but not the deeper sill-shaped source.

To corroborate our hypothesis, we performed a quantitative numerical model of the interaction between the plumbing systems of Mauna Loa and Kīlauea. The Figs. 7 and S13 in supplementary material show the volumetric strain and stress fields as the consequence of internal overpressure applied to each source independently. We selected the overpressure values for each source to reproduce approximately the maximum observed ground deformation above each source. Figure 7 shows the volumetric strain produced by a pressure change inside the Mauna Loa dike-shaped source (see panel (C) in Fig. 7). It can be seen that the strain field in the surroundings of the shallow source at Kīlauea is perturbed. The Mauna Loa Okada source does not significantly affect the deeper Kilauea reservoir. Furthermore, panels (D) and (E) in Fig. 7 show that pressure changes inside the Kīlauea sources does not significantly affect Mauna Loa. Therefore we conclude that the interaction through stress-transfer is effective only between the Mauna Loa dike with respect to the shallow Mogi-shaped Kīlauea reservoir. In the supplementary Fig. S13, we also show the corresponding isotropic component of the perturbed stress field.

## Conclusions

The application of ICA decomposition to four DInSAR SBAS datasets revealed an anticorrelated behaviour between Mauna Loa and Kīlauea volcanoes during the studied interval 2003–2010. At the same time, another pattern of ground deformation has been identified and is linked to independent behaviour at Kīlauea alone. The GPS dataset and inverse modelling results support these findings. Moreover, another significant result from our analysis is the evidence of a single ground deformation source at Mauna Loa during the studied time interval. This has been previously suggested by Pepe et al.^5^, who showed the central conduit dynamics and the dikes along the rift zones displayed an almost synchronous inflation. Kīlauea displays a greater complexity, with at least two sources simultaneously active.

We highlight that the connection between Mauna Loa and Kīlauea occurs at shallow depths in the first few kilometers of the crust, through a stress transfer mechanism. Stress transfer at Mauna Loa and Kīlauea has been considered by various authors to explain the dynamics of intrusions along rift zones^14,18^ and the interaction between earthquakes and eruptions^4,5^ at both volcanoes. This interconnection is created by the Mauna Loa reservoir perturbing the Kīlauea shallowest source. Conversely, the sources below the Kīlauea do not effectively influence the Mauna Loa reservoir. In practice, the inflation of Mauna Loa makes the stress field in the surroundings of Kīlauea less favourable for the ascent of magma into its shallow reservoir. The opposite mechanism, with Kīlauea affecting Mauna Loa, seems less favorable. The respective geometries of the sources (Fig. 7) make an effective mechanical interaction possible only between the dike-shaped source of Mauna Loa to the shallow volumetric source of Kīlauea. The sill-shaped geometry of the deeper Kīlauea source means it is less affected by this interaction process, as also confirmed by numerical modelling.

An important result of this work is the application of ICA to ground deformation datasets. This statistical tool has been demonstrated to effectively detect and separate individual independent sources within complex spatio-temporal ground deformation patterns. This approach greatly simplifies the study of complex ground deformation sources, whose components can be modelled independently. Using more advanced inverse modelling tools^5^ can shed further light on the spatial complexity of the Kīlauea plumbing system. Further studies should be devoted to analyzing the interaction between ground deformation sources over a larger time span.

## Methods

### DInSAR SBAS time series

In this work, was utilised the large amount of satellite data acquired over eight years (2003–2010) across Hawai’i Island. Specifically, C-band (wavelength of $\approx$ 5.6 cm) ASAR ENVISAT images from the European Space Agency (ESA) were acquired along both ascending and descending orbits from 2003-2010 (see Fig. S3 in the supplementary material). Four tracks were selected: T093 along an ascending orbit, and T343, T472, and T200 (Fig. S3 in supplementary material) along descending orbits. The tracks have different swathes: I2, I7, I1, and I2, respectively (see Table S2 for tracks details). We analysed a catalog of 394 SAR images covering both Mauna Loa and Kīlauea volcanoes, with look angles ranging from $15.0^{\circ }$ (I1) to $45.2^{\circ }$ (I7)^5^. All the interferograms of the considered tracks were analysed automatically, and noisy interferograms were discarded. We performed a multitemporal analysis using the Small Baseline (SBAS) technique that provided a Line-Of-Sight (LOS) time series for coherent pixels of the SAR images. SBAS algorithms allow the production of a deformation time series for each coherent pixel^34^. For data processing, we employed the Grid Processing On-Demand (G-POD) platform of ESA that applies the SBAS algorithm and can process large volumes of DInSAR data^35^. Tracks covering only one of the two volcanoes were discarded from the analysis (see Figs. S4, S5, S6, and Table S4 in the supplementary material).

### Independent component analysis (ICA) of DInSAR SBAS time series

Independent Component Analysis is a multivariate statistical tool that allows the separation of a spatio-temporal dataset into discrete components, for which the relative statistical independence is maximised^36^. ICA is a widely recognized technique that allows the detection of “hidden” patterns in complex datasets^36^. An advantage of using ICA in volcano geodesy is that it allows simplification of the inverse problem by separating the contribution of different causative ground deformation sources. ICA was highlighted by Ebmeier^37^ as a valuable tool in the detection of different sources responsible for observed ground deformation patterns^1^. For Hawai’i Island, each track dataset consists of a set of time series $L_(xi, tj)$, where L is the LOS displacement for a given track, $xi$ is the spatial position of the i-th DInSAR pixel, $tj$ is the time of the j-th DInSAR image.

Using ICA, the original dataset can be decomposed into a finite sum of N components with a fixed spatial pattern $B_(xi)$ and time-varying amplitudes. In practice, the observed LOS displacement time series for a given track can be represented as:

1 $$\begin{aligned} L(i,t_{j}) = \sum _{k=1}^{N} A_{jk} B_k (x_i), \end{aligned}$$

where $B_k$ is the normalised spatial pattern corresponding to the k-th ICA component, and $A_{jk}$ is the amplitude of the k-th ICA component at time $t_{j}$.

In the model of Eq. (1), the LOS displacement is expressed through a linear combination of the normalised spatial patterns $B_{k}$, through the time-varying coefficients $A_{jk}$. Once the $B_{k}$ are known from ICA, the coefficients $A_{jk}$ can be determined by solving the linear system of Eq. (1) using a least-squares approach. This model implies that using only a limited number of components, as is usual when performing ICA, the sum on the right side of Eq. (1) cannot fully model the whole signal L. If the number of selected components is sufficient, this missing value is generally related only to the incoherent noisy part of the signal.

The ICA components can be ordered based on their energy, defined as the sum of squared $A_{jk}$ for each $k$. This allows a consistent sorting of the retrieved components, irrespective of the specific ICA algorithm used. In our case, using three components was sufficient since additional components had negligible amplitude values.

The first and the second components (ICA1, ICA2) were associated with a clear and meaningful signal, while the third component (ICA3) was mainly composed of noise; hence it was not considered further. In Table S3 (in supplementary material) we show the percentage of the energy of the ICA signal in every component for each track of the study. It should be noted that for the purposes of ICA decomposition, it is recommended to have a minimum of 3 components so that at least one of them includes the noisy part of the signal^1,36,37^.

### Non-linear inverse modelling

To model the observed ground deformation, we tested four analytical source models^33^: the Mogi point source^31^, the spheroidal source^38^, the closed pipe^39^ and the rectangular crack^32^. In all cases, we performed the modelling assuming a Poisson’s ratio of 0.25 within a half-space. We selected the best model for each ICA and each volcano following the Akaike Information Criterion (AIC)^30^. We performed a non-linear inversion of each track for all previous source models for each ICA and each volcano. The inverse method relies on a non-linear optimisation of a misfit function using the Nelder-Mead simplex algorithm^40^. The misfit function is defined as the sum of the squared residuals between the observed and synthetic data. The synthetic data, computed using the aforementioned analytical models, were projected along the LOS corresponding to each track. The final models resulted from averaging the results obtained for each track. We obtained a source model for Mauna Loa, corresponding to ICA1, and two models for Kīlauea, corresponding to ICA1 and ICA2, respectively.

### GPS data

GPS stations up to 10 km from the summit calderas of the two volcanoes were selected (Fig. S3 in supplementary material). For Mauna Loa and Kīlauea volcanoes, 8 and 7 stations were selected, respectively, with data from 2008 to 2011. The GPS daily solutions were downloaded from the Nevada University repository (http://geodesy.unr.edu)^41^.

We used horizontal components to compare results obtained through the ICA decomposition of DInSAR SBAS data with GPS time series, since vertical components have a higher signal/noise ratio. The areal strain time series was computed since it does not require the assumption of a reference point on the island. The areal strain is a geodetic method widely applied in active volcanic areas and has been described by many authors^42–45^. For this purpose, we computed the area for triplets of GPS stations covering more than half of the summit calderas (see Figs. S7, S8 in supplementary material). We did not use triplets with significant temporal data gaps or whose signal was too noisy. Accordingly, we used 11 triplets for the main crater of Mauna Loa and 15 triplets for Kīlauea’s summit caldera. We studied the temporal variation in the area of a given triplet of stations, determining the areal strain time series $\delta A(t)$ as:

2 $$\begin{aligned} \delta A(t) = (A(t_{0}) - A\frac{(t)}{A}(t_0)), \end{aligned}$$

with $\delta A(t)$ being the times series of the area of a given triplet and $A(t_{0})$ the area at the initial time of the series. We did not consider the detailed spatial variations of the areal strain since we are making only a qualitative comparison of the trend of DInSAR SBAS and areal strain data here. Most of the GPS stations at Kīlauea began operation in the first quarter of 2008. Hence, we computed the areal strain time series for this volcano beginning in mid-2008, when the GPS network was fully operative. Therefore, we also begin the comparison with the Mauna Loa time series from June 2008 (although more data is available).

### Numerical modelling of the stress and strain fields

To validate the stress-transfer model, we realized a finite-element tridimensional elastic modelling using the software COMSOL Multiphysics ®. The model includes the topography of Hawaii Island and the sources of the ground deformation determined by non-linear inversion. The size of the computational domain was 100x100x70 $\mathrm{km}^3$. The linear elastic material characteristics were assumed as isotropic, with elastic constants retrieved from the 1D velocity model of Lin et al.^46^. The boundary conditions of the domain were chosen to be fixed on the bottom and lateral sides. The computational domain was built as tetrahedral mesh elements with dimensions ranging between 150 and 3500 m. As explained in the “Discussion” section, we applied an overpressure to each source, calculating the corresponding perturbation of the stress and strain fields.

## Supplementary Information

Supplementary Information.

## Data Availability

The DInSAR SBAS datasets used and/or analysed during the current study available from the corresponding author on reasonable request. The GPS daily solutions were downloaded from the Nevada University repository (http://geodesy.unr.edu).
