# Supplementary material for: Elastic interaction between Mauna Loa and Kīlauea evidenced by independent component analysis
Source: Sci Rep. 2022 Nov 18;12:19863. doi: 10.1038/s41598-022-24308-0 (PMC9674672; doi:10.1038/s41598-022-24308-0)
Supplement: Supplementary file 1 — Supplementary Information. [file 41598_2022_24308_MOESM1_ESM.pdf]

Supplementary materials for the article "Elastic interaction between Mauna Loa and Kīlauea evidenced by Independent Component Analysis".

Monika Przeor (1,2), Luca D'Auria (1,2), Susi Pepe (3), Pietro Tizzani (3) and Iván Cabrera-Pérez (2)

1 Instituto Tecnológico y de Energías Renovables (ITER), Granadilla de Abona, Spain
2 Instituto Volcanológico de Canarias (INVOLCAN), San Cristóbal de La Laguna, Spain
3 Istituto per il Rilevamento Elettromagnetico dell'Ambiente (IREA-CNR), Napoli, Italy
Corresponding author: mprzeor@iter.es; Tel.: +34-822-909-250 (M.P.)

List of Figures:

Figure S1 shows the areal strain series for Mauna Loa GPS stations.

Figure S2 shows the areal strain series for Kīlauea GPS stations.

Figure S3 shows the location of satellite tracks and GPS stations.

Figure S4 shows the location of additional ENVISAT satellite tracks.

Figure S5 shows the LOS deformation maps, ICA1, and ICA2 of Mauna Loa.

Figure S6 shows the LOS deformation maps, ICA1, and ICA2 of Kīlauea.

Figure S7 shows the Mauna Loa GPS station triplets used for computing the areal strain time series.

Figure S8 shows the Kīlauea GPS station triplets used for computing the areal strain time series.

Figure S9 shows the LOS deformation cumulative maps, first and second component decomposition of ENVISAT 093 orbit for different time intervals.

Figure S10 shows the LOS deformation cumulative maps, first and second component decomposition of ENVISAT 343 orbit for different time intervals.

Figure S11 shows the LOS deformation cumulative maps, first and second component decomposition of ENVISAT 472 orbit for different time intervals.

Figure S12 shows the LOS deformation cumulative maps, first and second component decomposition of ENVISAT 200 orbit for different time intervals.

Figure S13 shows a 3D numerical modelling of stress fields of each source of the ground deformation.

List of Tables:

Table S1 shows the parameters of modelled sources of ground deformation.

Table S2 shows the inventory of the four analysed ENVISAT satellite data tracks in the 2003-2010 period, covering both volcanoes.

Table S3 shows the percentage of the energy of every component of decomposition for each track.

Table S4 shows the inventory of the 6 analysed and discarded tracks of ENVISAT satellite data in the 2003-2010 period.

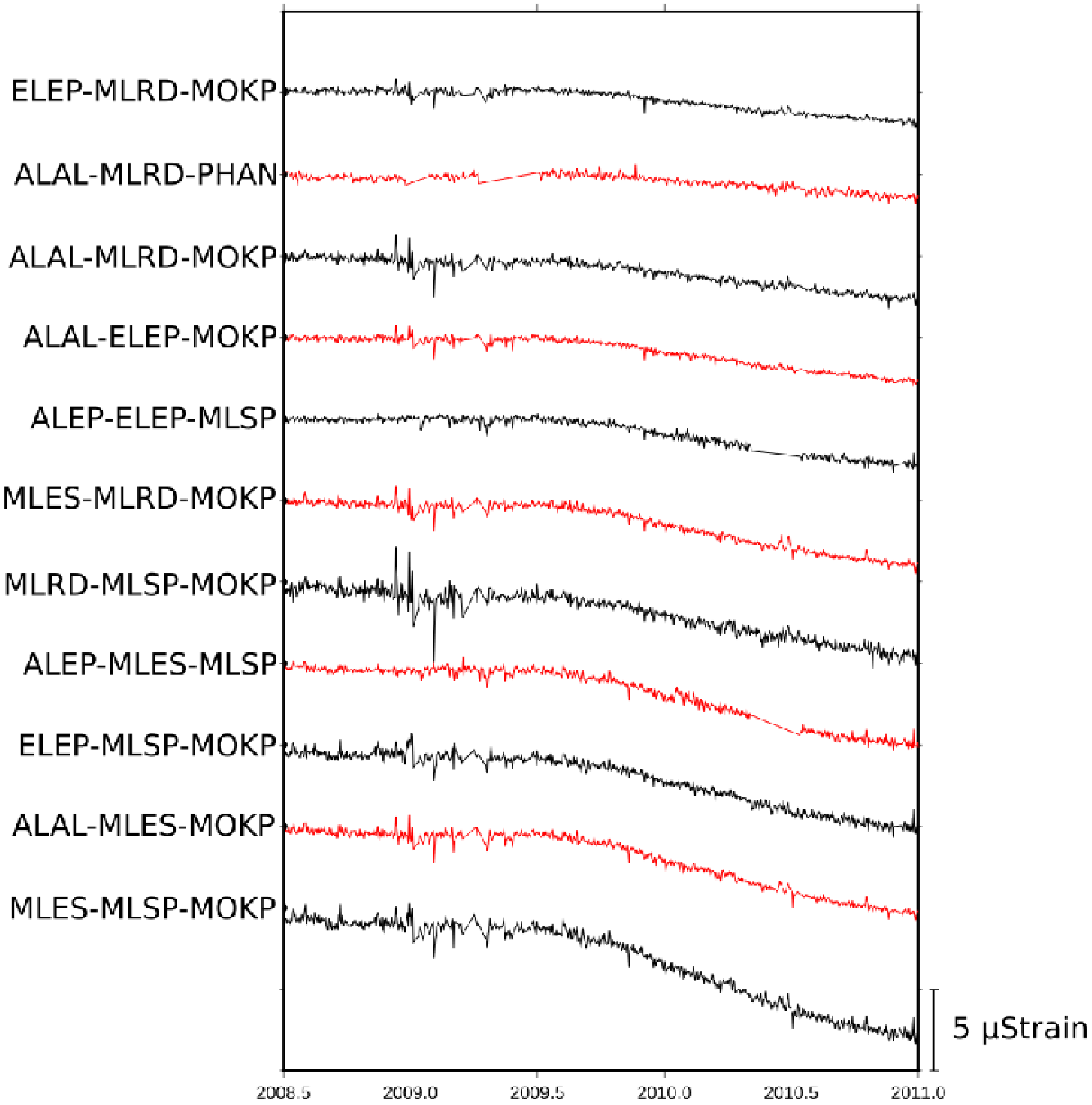

Figure S1. Areal strain time series for Mauna Loa GPS stations. The labels are triplets of GPS stations surrounding the summit cone of Moku‘ āweoweo Caldera (see Figure S2 for locations). The y-axis on the right shows the aerial strain scale in μstrain, the dimension of the vertical axis is 5 μstrain, Time series colours are alternated in black and red.

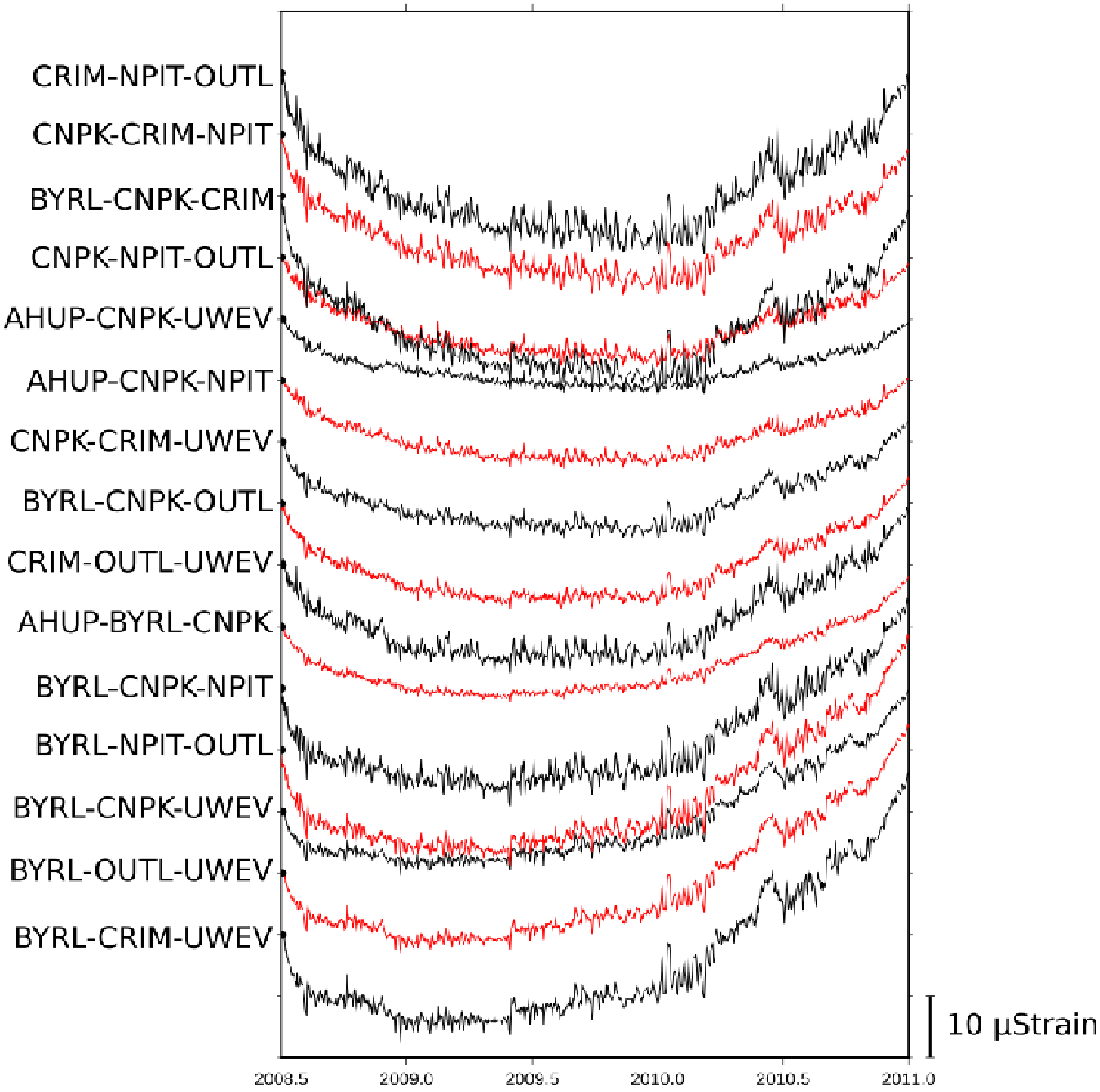

Figure S2. Areal strain time series for Kīlauea GPS stations. The labels are triplets of GPS stations surrounding the summit cone of Halemaʻumaʻu Crater (see Figure S2 for the locations). The y-axis on the right shows the aerial strain scale in µstrain, the dimension of the vertical axis is 10 µstrain, being marked by ticks. Note that the axis scale in this figure is different from that of Figure S1. Time series colours are alternated in black and red.

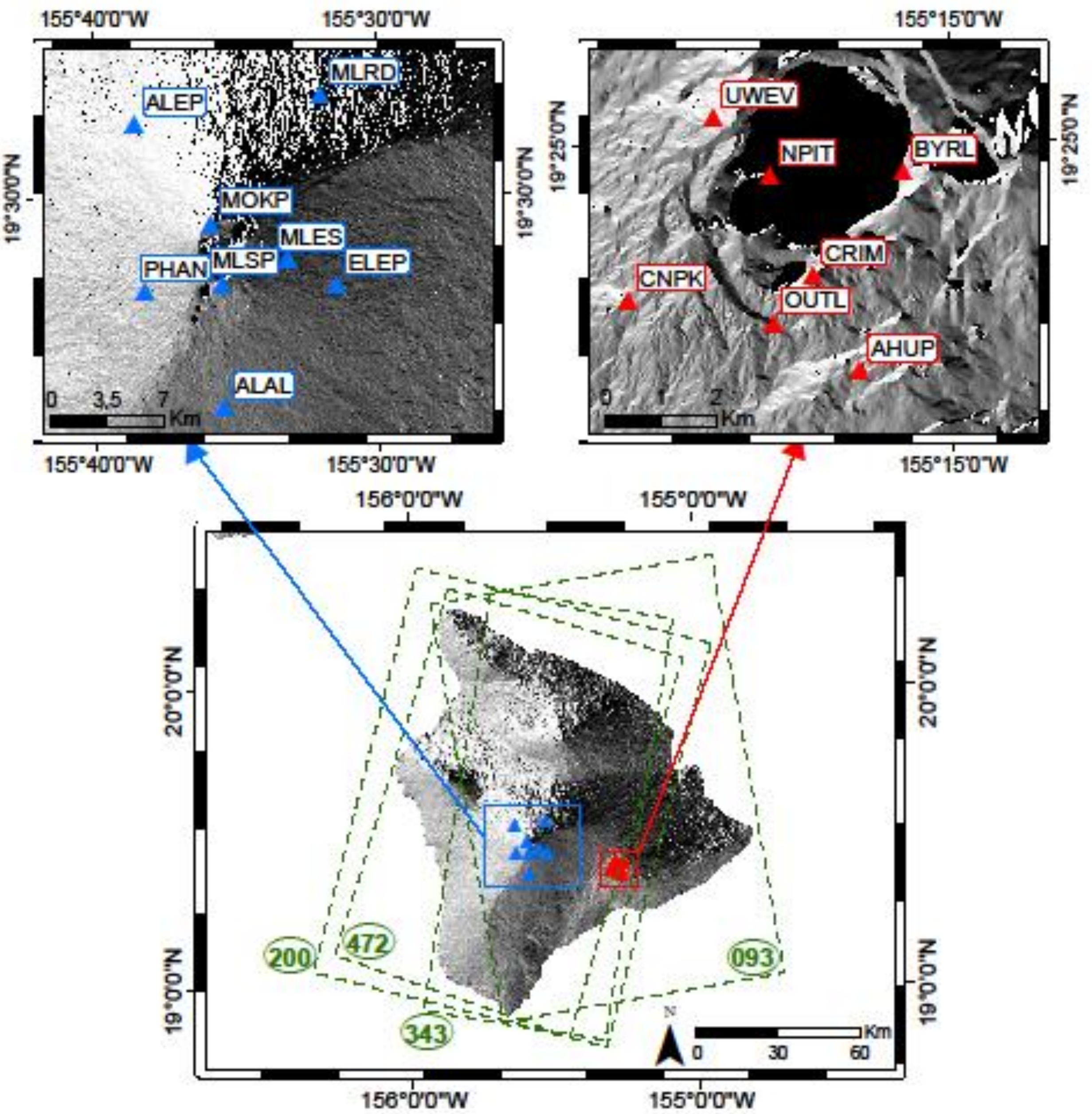

Figure S3. Location of satellite tracks and GPS stations. The lower map shows Hawai`i Island. The blue rectangle shows the GPS study area of Mauna Loa, while the red rectangle represents the area of Kīlauea GPS stations. Green rectangles mark ENVISAT satellite data tracks along ascending (093) and descending orbits (343, 472, and 200). The upper maps show Mauna Loa and Kīlauea summits, respectively, with blue triangles representing the GPS stations of Mauna Loa and red triangles, GPS stations of Kīlauea.

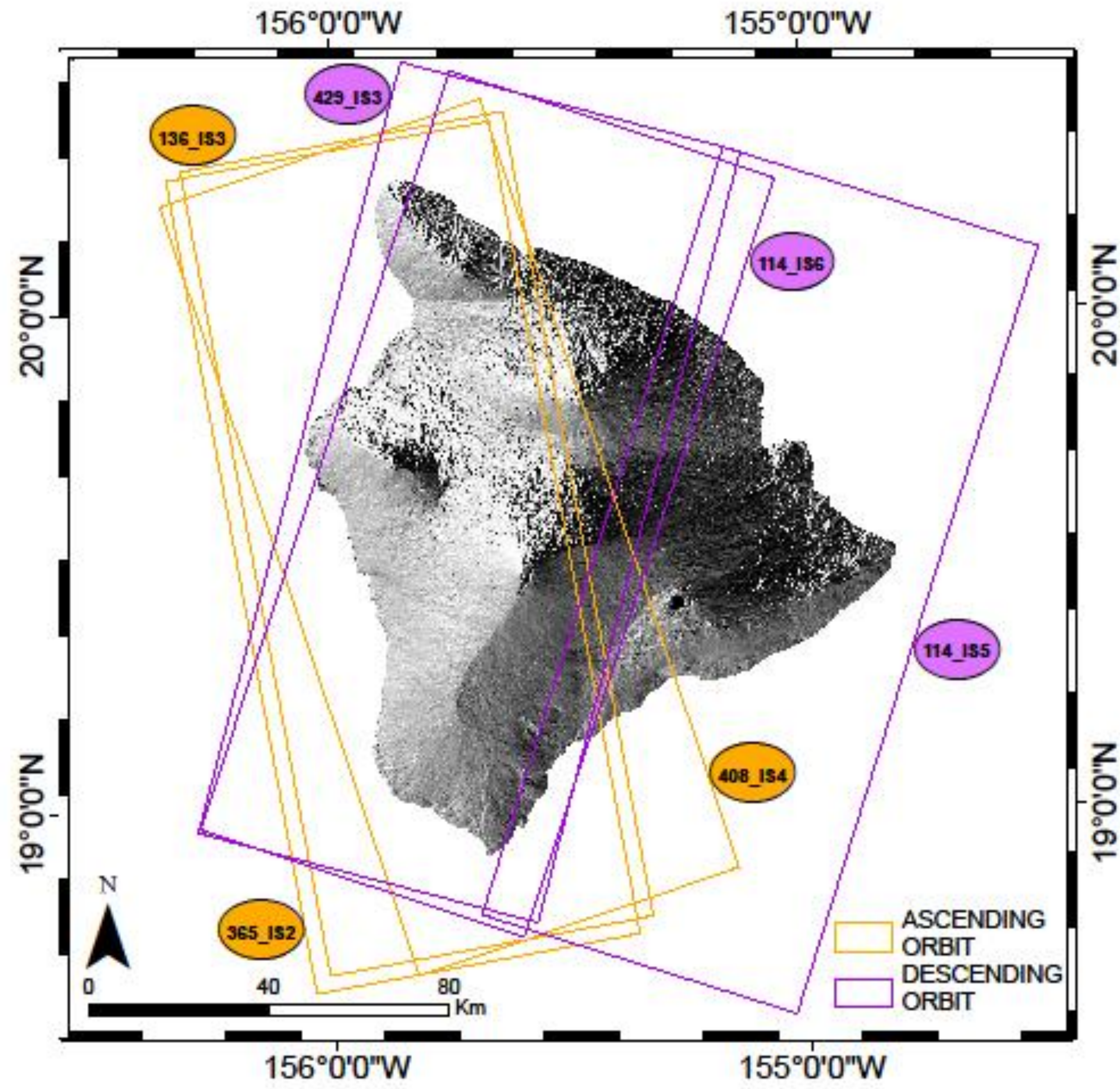

Figure S4. Location of additional ENVISAT satellite tracks. Purple rectangles mark the tracks of ENVISAT satellite along descending orbits with swaths varying from I3 to I6 (26.0° to 42.8°); orange rectangles mark the tracks of ENVISAT satellite along ascending orbits with swaths varying from I2 to I4 (19.2° to 36.3°).

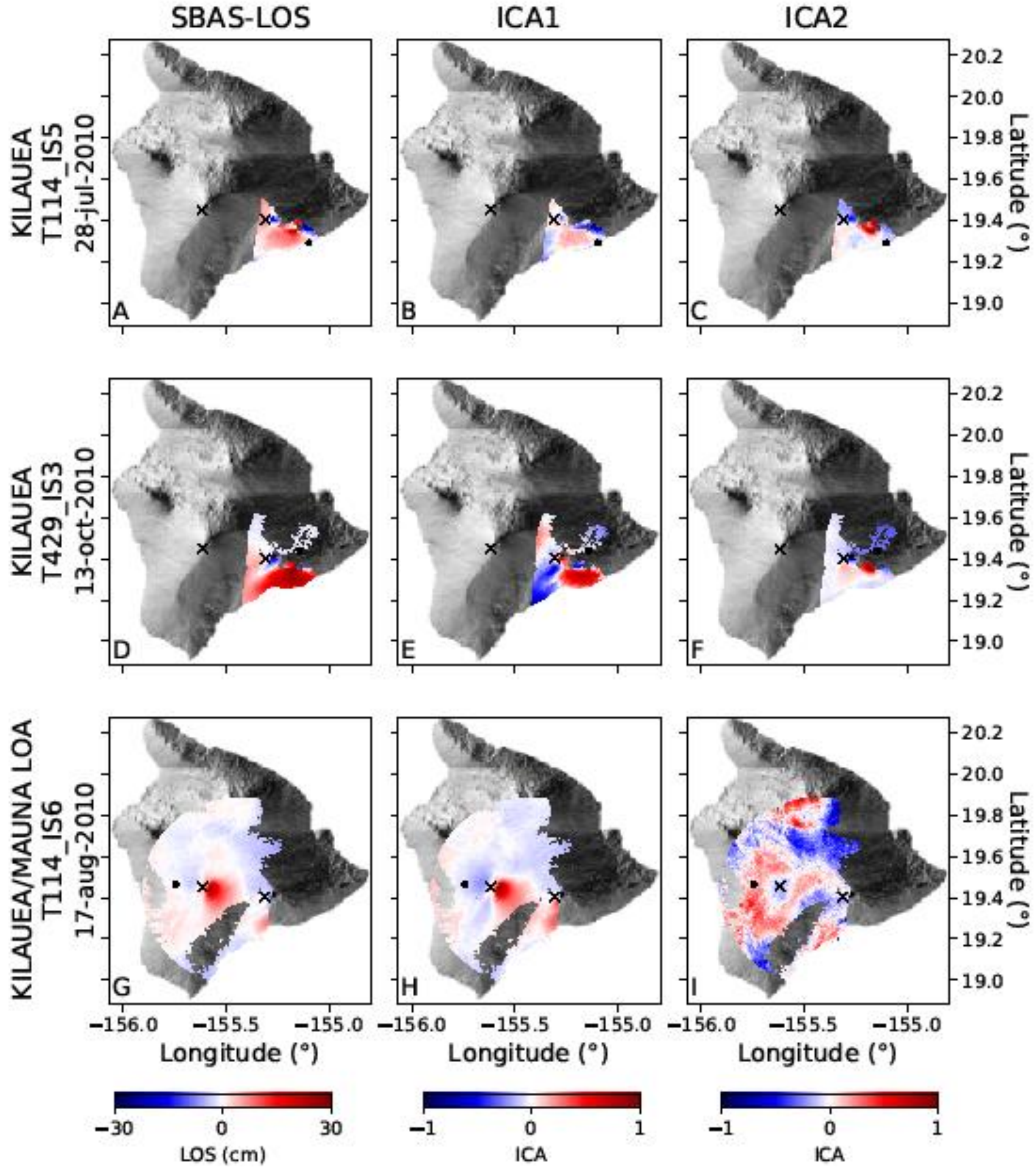

Figure S5. LOS deformation maps of Mauna Loa. A,B,C: ENVISAT 136 (IS3) orbit; D,E,F: ENVISAT 365 (IS2) orbit; G,H,I: ENVISAT 408 (IS4) orbit (see table S4 for track details). Column 1 shows raw data; columns 2 and 3 represent respectively the first and the second component resulting from applying the ICA decomposition algorithm. Black crosses: see figure 2 for description, while black dots are the reference points used to the SBAS-LOS performance.

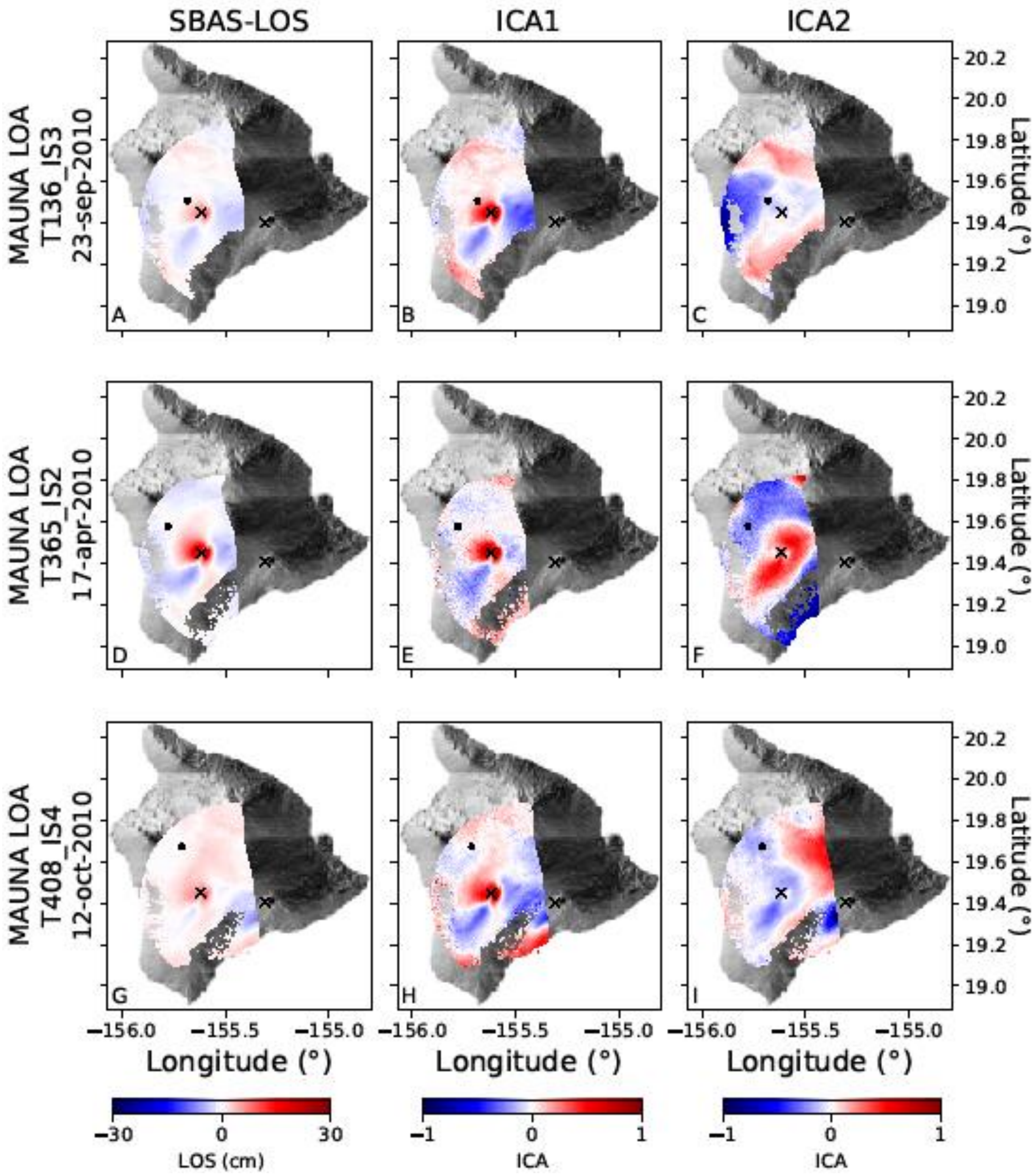

Figure S6. LOS deformation maps of Kīlauea. A,B,C: ENVISAT 114 (IS5) orbit; D,E,F: ENVISAT 429 (IS3) orbit; G,H,I: ENVISAT 114 (IS6) orbit (see table S1 for track details). Column 1 shows raw data; columns 2 and 3 represent respectively the first and the second component resulting from applying the ICA decomposition algorithm. Black crosses: see figure 2 for description, while black dots are the reference points used to the SBAS-LOS performance.

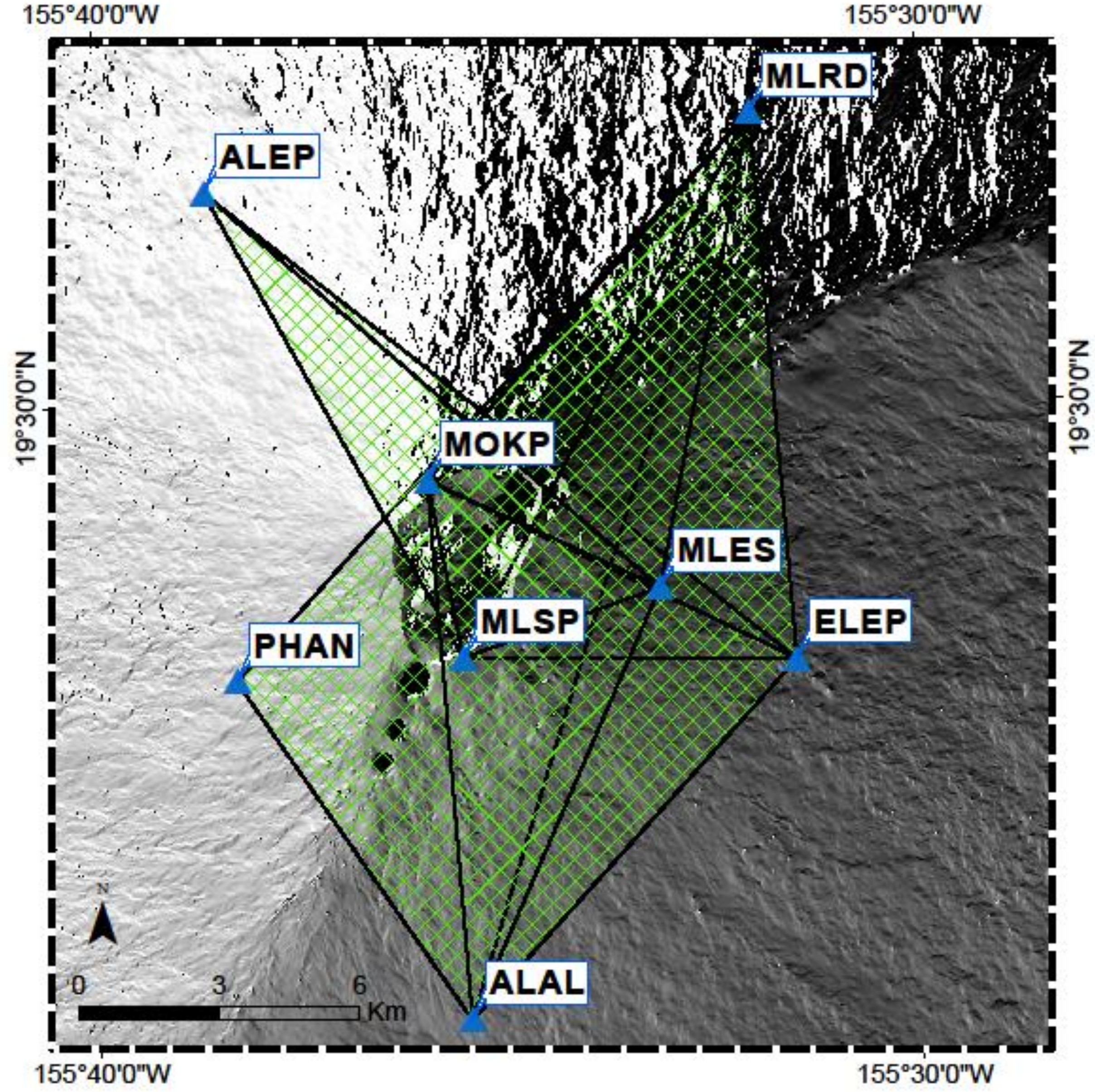

Figure S7. Mauna Loa GPS station triples used for computing the areal strain time series. Blue triangles represent the GPS stations on Mauna Loa summit Caldera. Every triplet of stations is bordered by black lines and filled by a green grid.

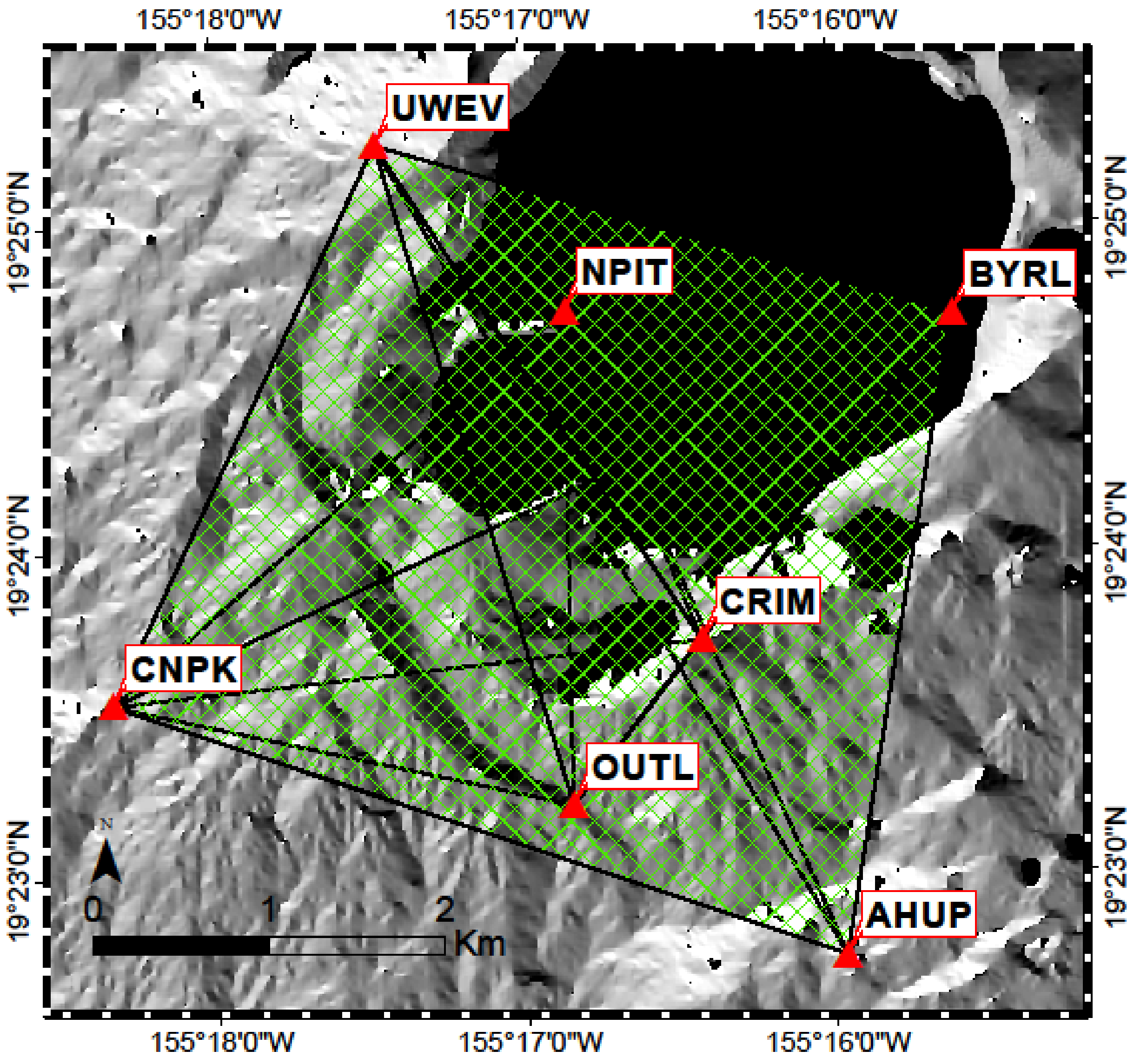

Figure S8. Kīlauea GPS station triples used for computing the areal strain time series. Red triangles represent the GPS stations in Kīlauea summit Caldera. Every triplet of stations is bordered by black lines and filled by a green grid.

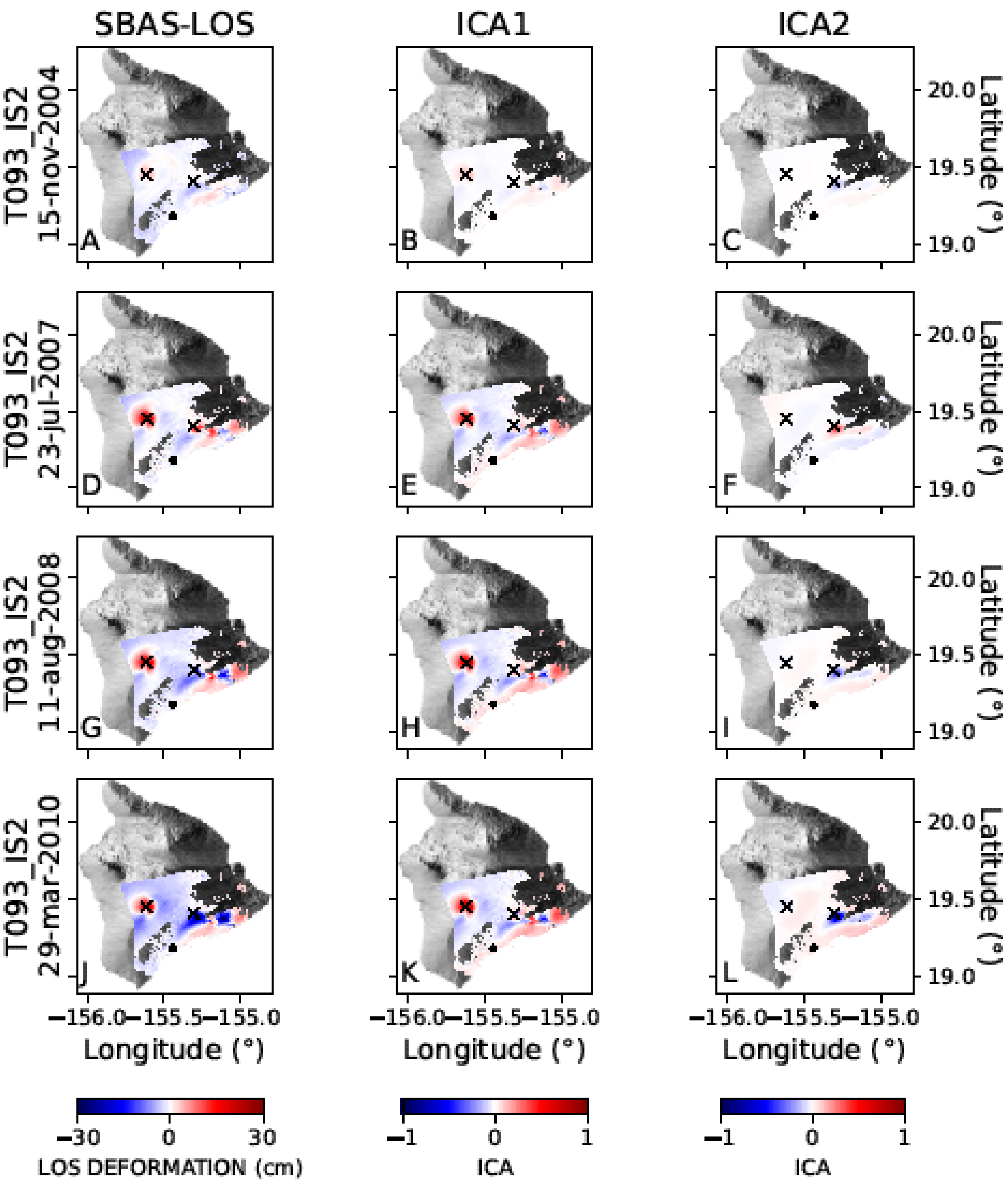

Figure S9. LOS deformation cumulative maps, first and second component of ICA decomposition of ENVISAT 093 orbit for four different intervals. A, D, G, J: LOS cumulative displacement DInSAR map; B, E, H, J (column 2) and C, F, I, L: (column 3) represent respectively the first and the second component resulting from applying the ICA decomposition algorithm. The black crosses indicate the points used for extracting the time series shown in figure 4, while black dots are the reference points used for the DInSAR SBAS processing.

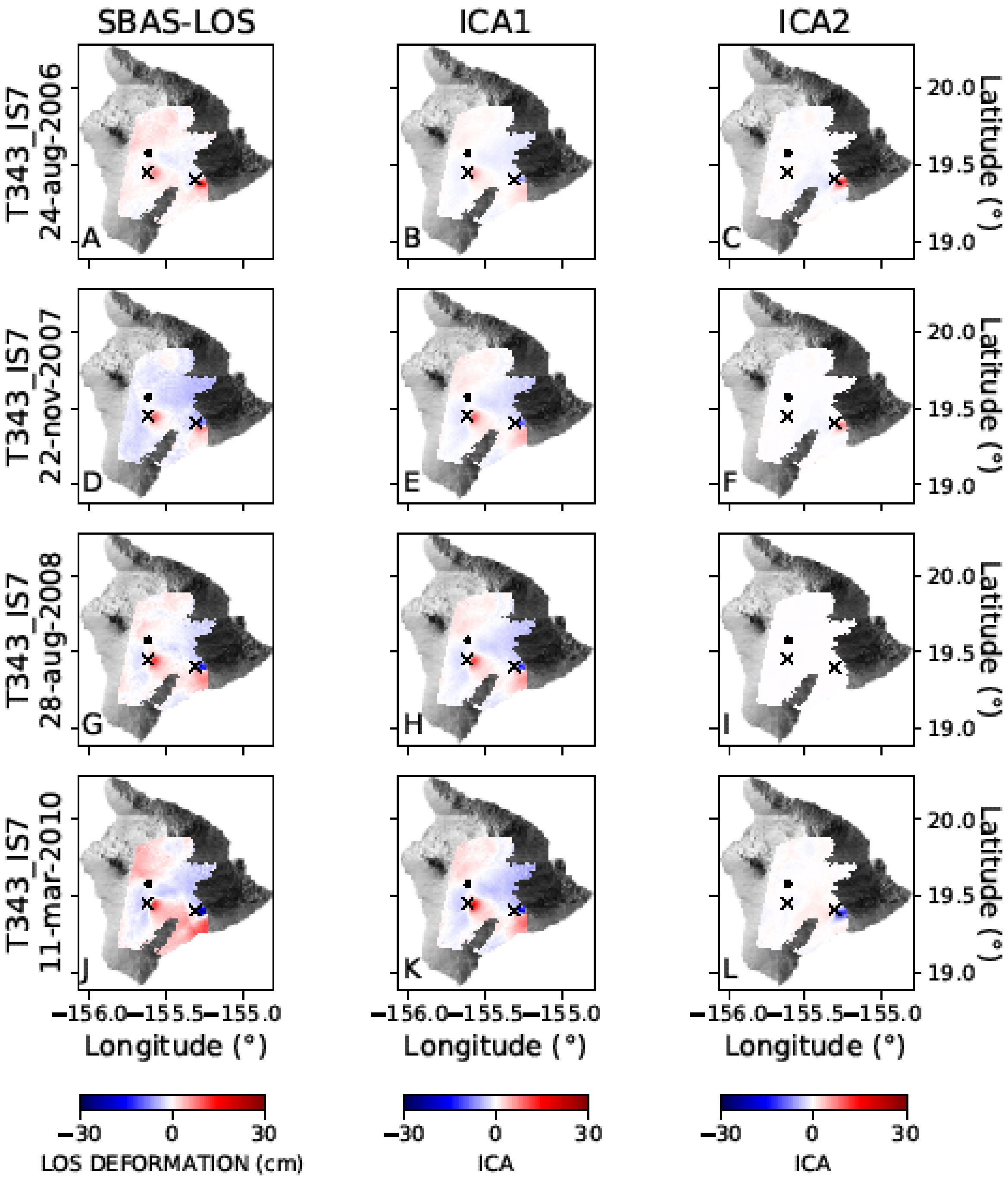

Figure S10. LOS deformation cumulative maps, first and second component of ICA decomposition of ENVISAT 343 orbit for four different intervals. A, D, G, J: LOS cumulative displacement DInSAR map; B, E, H, J (column 2) and C, F, I, L: (column 3) represent respectively the first and the second component resulting from applying the ICA decomposition algorithm. The black crosses indicate the points used for extracting the time series shown in figure 4, while black dots are the reference points used for the DInSAR SBAS processing.

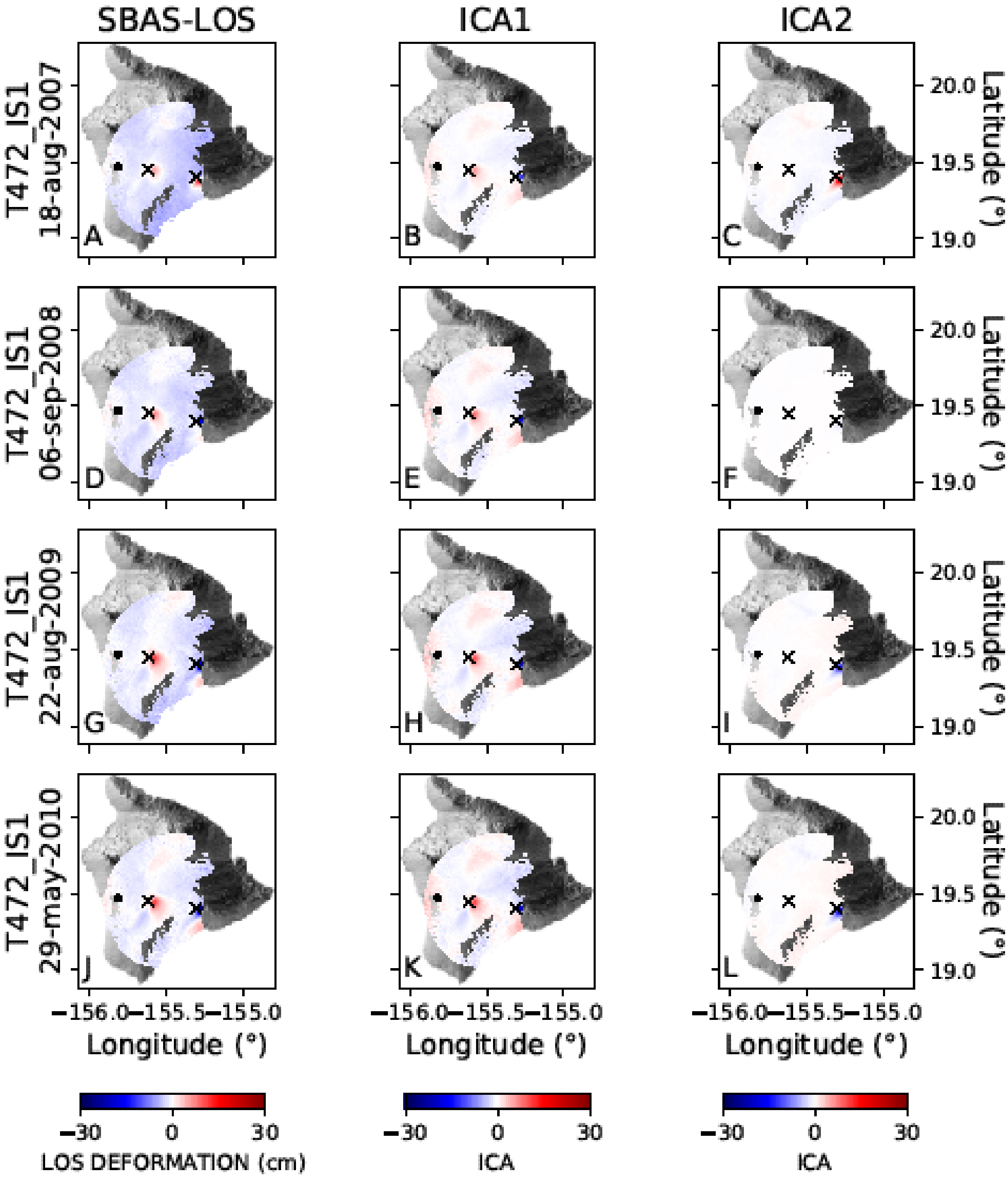

Figure S11. LOS deformation cumulative maps, first and second component of ICA decomposition of ENVISAT 472 orbit for four different intervals. A, D, G, J: LOS cumulative displacement DInSAR map; B, E, H, J (column 2) and C, F, I, L: (column 3) represent respectively the first and the second component resulting from applying the ICA decomposition algorithm. The black crosses indicate the points used for extracting the time series shown in figure 4, while black dots are the reference points used for the DInSAR SBAS processing.

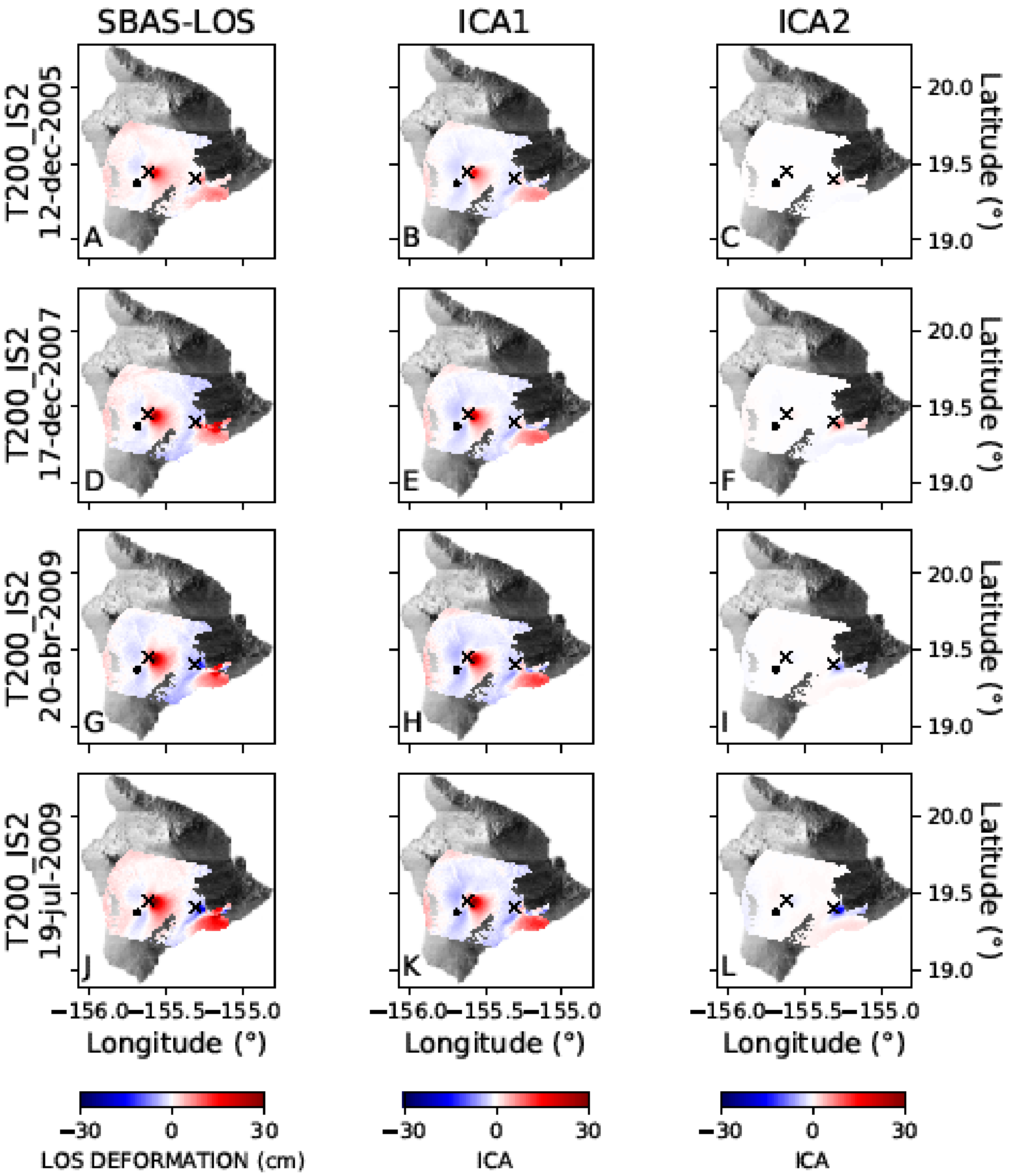

Figure S12. LOS deformation cumulative maps, first and second component of ICA decomposition of ENVISAT 200 orbit for four different intervals. A, D, G, J: LOS cumulative displacement DInSAR map; B, E, H, J (column 2) and C, F, I, L: (column 3) represent respectively the first and the second component resulting from applying the ICA decomposition algorithm. The black crosses indicate the points used for extracting the time series shown in figure 4, while black dots are the reference points used for the DInSAR SBAS processing.

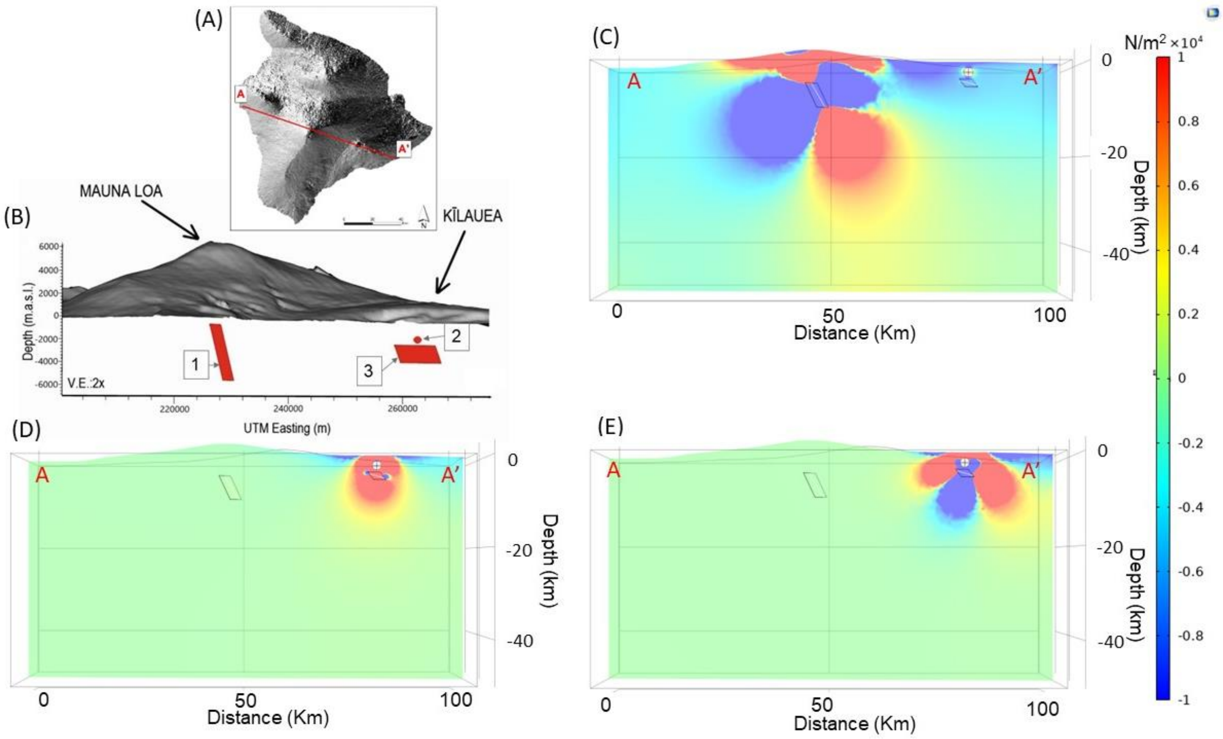

Figure S13. 3D numerical modelling of stress fields. (A) The Digital Elevation Model of Hawai'i Island, with the red line representing the trace (A-A'), used in panels (C-E). (B) tridimensional representation of the modelled ground deformation sources of Mauna Loa and Kīlauea volcanoes. The sources are numbered according to Table S1 in the supplementary material. 1) the Mauna Loa dike-shaped source from ICA1; 2) the Kīlauea Mogi-like source from ICA1; 3) the Kīlauea sill-shaped source from ICA2. Storage areas and topography are exaggerated in size for clarity. (C) stress field represented along the A-A' trace due to an overpressure applied to the Mauna Loa dike-like source. (D) stress field along the A-A' trace due to an overpressure applied to the Kīlauea Mogi-like source. (E) stress field along the A-A' trace due to an overpressure applied to the Kīlauea sill-like source.

Table S1. Parameters of modelled sources of ground deformation. The results are the mean value and standard deviation of the modelled parameters of separated inversions of each track.

Source		Lat. (ºN)	Long. (ºW)	Depth (m)	Azimuth (º)	Dip (º)	Length (m)	Width (m)
**Mauna Loa (ICA1) OKADA**	Mean	19.43	-155.58	6204.6	28.2	62.1	3982.1	5572.4
	Std	0.009	0.015	1870.1	8.9	54.3	3646.1	4396.2
**Kīlauea (ICA1) MOGI**	Mean	19.40	-155.26	1202.5	X	X	X	X
	Std	0.011	0.008	471.9	X	X	X	X
**Kīlauea (ICA2) OKADA**	Mean	19.40	-155.26	3566.6	67.0	29.8	7708.1	3136.1
	Std	0.012	0.03	560.3	17.6	14.1	3577.8	2941.8

Table S2. Inventory of the four analysed ENVISAT satellite data tracks in the 2003-2010 period, covering both volcanoes.

TRACK	SWATH	LOOK ANGLE	ORBIT	START TIME	STOP TIME
**093**	IS2	19.2º-26.7º	ASCENDING	20/01/2003	07/06/2010
**343**	IS7	42.5º-45.2º	DESCENDING	17/11/2005	07/10/2010
**472**	IS1	15.0º-22.9º	DESCENDING	31/12/2005	11/09/2010
**200**	IS2	19.2º-26.7º	DESCENDING	08/12/2003	27/09/2010

Table S3. Percentage of energy of every component of decomposition for each track.

	TRACK 093	TRACK 343	TRACK 472	TRACK 200
**Component 1**	75.30%	82.30%	63.10%	84.20%
**Component 2**	19.40%	14.70%	32.60%	8.20%
**Component 3**	5.30%	2.90%	4.30%	7.60%

.

Table S4. Inventory of the 6 analyzed and discarded tracks of ENVISAT satellite data in 2003-2010 period.

TRACK	SWATH	LOOK ANGLE	ORBIT	START TIME	STOP TIME
**114**	IS5	35.8°-39.4°	DESCENDING	16/11/2004	28/07/2009
**429**	IS3	26.0°-31.4°	DESCENDING	12/02/2003	13/10/2010
**136**	IS3	26.0°-31.4°	ASCENDING	27/01/2005	23/09/2010
**365**	IS2	19.2°-26.7°	ASCENDING	08/02/2003	17/04/2010
**408**	IS4	31.0°-36.3°	ASCENDING	27/12/2005	12/10/2010
**114**	IS6	39.1°-42.8°	DESCENDING	07/08/2005	17/08/2010
